# Physiotherapeutic and non-conventional approaches in patients with chronic low-back pain: a level I Bayesian network meta-analysis

**DOI:** 10.1038/s41598-024-62276-9

**Published:** 2024-05-21

**Authors:** Alice Baroncini, Nicola Maffulli, Luise Schäfer, Nicola Manocchio, Michela Bossa, Calogero Foti, Alexandra Klimuch, Filippo Migliorini

**Affiliations:** 1 GSpine4, IRCCS Ospedale Galeazzi – Sant’Ambrogio, Milano, Italy; 2 Department of Orthopaedics, Faculty of Medicine and Psychology, University of Rome “La Sapienza”, Rome, Italy; 3 School of Pharmacy and Bioengineering, Faculty of Medicine, Keele University, Stoke on Trent, ST4 7QB UK; 4 Centre for Sports and Exercise Medicine, Barts and the London School of Medicine and Dentistry, Mile End Hospital, Queen Mary University of London, London, E1 4DG UK; 5 Department of Orthopaedic, Trauma, and Reconstructive Surgery, RWTH University Medical Centre, 52074 Aachen, Germany; 6 Physical and Rehabilitation Medicine, Clinical Sciences and Translational Medicine Department, Tor Vergata University, Rome, Italy; 7 Department of Orthopedics and Trauma Surgery, Academic Hospital of Bolzano (SABES-ASDAA), Teaching Hospital of the Paracelsus Medical University, 39100 Bolzano, Italy

**Keywords:** Physical, Therapy, Conservative, Pain, Medical research, Outcomes research

## Abstract

Chronic low back pain (cLBP) is a major cause of disability and healthcare expenditure worldwide. Its prevalence is increasing globally from somatic and psychosocial factors. While non-pharmacological management, and in particular physiotherapy, has been recommended as a first-line treatment for cLBP, it is not clear what type of physiotherapeutic approach is the most effective in terms of pain reduction and function improvement. This analysis is rendered more difficult by the vast number of available therapies and a lack of a widely accepted classification that can effectively highlight the differences in the outcomes of different management options. This study was conducted according to the PRISMA guidelines. In January 2024, the following databases were accessed: PubMed, Web of Science, Google Scholar, and Embase. All the randomised controlled trials (RCTs) which compared the efficacy of physiotherapy programs in patients with cLBP were accessed. Studies reporting on non-specific or mechanical cLPB were included. Data concerning the Visual Analogic Scale (VAS) or numeric rating scale (NRS), Roland Morris Disability Questionnaire (RMQ) and Oswestry Disability Index (ODI). Data from 12,773 patients were collected. The mean symptom duration was 61.2 ± 51.0 months and the mean follow-up was 4.3 ± 5.9 months. The mean age was 44.5 ± 9.4 years. The mean BMI was 25.8 ± 2.9 kg/m^2^. The Adapted Physical Exercise group evidenced the lowest pain score, followed by Multidisciplinary and Adapted Training Exercise/Complementary Medicine. The Adapted Physical Exercise group evidenced the lowest RMQ score followed by Therapeutic Exercises and Multidisciplinary. The Multidisciplinary group evidenced the lowest ODI score, followed by Adapted Physical Exercise and Physical Agent modalities. Within the considered physiotherapeutic and non-conventional approaches to manage nonspecific and/or mechanic cLBP, adapted physical exercise, physical agent modalities, and a multidisciplinary approach might represent the most effective strategy to reduce pain and disability.

## Introduction

Chronic low back pain (cLBP) is one of the global leading causes of disability and healthcare expenditure^1–3^. First-ever episodes of LBP have an incidence of 15%, and 80% of subjects experience at least one episode of activity-limiting LBP within one year^4^. The prevalence of cLBP is increasing not only because of population ageing and obesity but also as a consequence of psychosocial and economic strains^5–7^. Thus, considerable efforts have been put in place to identify the most effective way to manage this condition^8–11^. Recent guidelines suggest non-pharmacologic treatment as first-line therapy, accompanied by pharmacologic management when symptoms cannot be sufficiently controlled^12–14^.

Physiotherapy has emerged as an effective and non-invasive approach for the management of cLBP, with the goal to improve pain and disability by acting on muscular strength and flexibility, range of motion, and muscular imbalance^15–17^. Furthermore, education and lifestyle modifications aim to provide patients with the tools to prevent future episodes of cLBP^18–21^. Different physiotherapeutic regimes have been developed and investigated in this setting^22,23^. In particular, different forms of exercise, manual therapy, physical agent modalities, and education, or a combination of these in a multidisciplinary approach have been efficiently applied in the setting of cLBP^24,25^. Available guidelines also highlight a discrepancy regarding the most effective physiotherapeutic management, and clear directions in this respect are lacking^13,26,27^. The available literature has focused on one particular type of physiotherapy at a time or has directly compared a limited number of similar approaches^28,29^. The lack of a widely accepted classification of the different physiotherapeutic management options has obviously made direct comparisons difficult. In particular, available classifications have failed to group physiotherapeutic approaches in a way that would allow to highlight possible outcome differences in terms of pain management and function improvement^30,31^.

This investigation compared the efficacy of the different physiotherapeutic and non-conventional approaches in the setting of nonspecific and/or mechanic cLBP. A Bayesian network meta-analysis of level I studies was conducted for this purpose.

## Methods

### Eligibility criteria

All the randomised controlled trials (RCTs) which compared the efficacy of conventional and non-conventional physiotherapy programs in patients with cLBP were accessed. According to the authors´ language capabilities, articles in English, German, Italian, French, and Spanish were eligible. Only RCTs with level I of evidence, according to the Oxford Centre of Evidence-Based Medicine^32^, were considered. Reviews, opinions, letters, and editorials were not considered. Animals, in vitro, biomechanics, computational, and cadaveric studies were not eligible. Studies reporting on non-specific^33^ or mechanical^34^, cLPB were included. The pain was defined as chronic when symptoms persisted for a minimum of three months^7^. Studies including patients with radiculopathy and/or neurologic symptoms were excluded from this analysis. Only studies which analysed patient-reported outcome measures (PROMs) were considered. Missing quantitative data under the outcomes of interest warranted the exclusion of the study.

### Search strategy

This study was conducted according to the 2015 PRISMA Extension Statement for Reporting of Systematic Reviews Incorporating Network Meta-Analyses of Health Care Interventions^35^. The following algorithm was established:

- P (Problem): cLBP;
- I (Intervention): Physiotherapy;
- C (Comparison): different modalities of physiotherapy;
- O (Outcomes): pain and disability.

In January 2024, the following databases were accessed: PubMed, Web of Science, and Embase. No time constraint was set for the search. The search was restricted to only RCTs. The medical subject headings (MeSH) used in PubMed are shown in the appendix. No additional filters were used in the database search.

### Selection and data collection

Two authors (A.K., L.S.) performed the database search. Disagreements were settled by a third author (N.M.) with long experience on systematic reviews. All the resulting titles were screened by hand and, if suitable, the abstract was accessed. If the abstract matched the topic, the full text was accessed. If the full text was not accessible or available, the article was not considered for inclusion. A cross reference of the bibliography of the full text was also conducted to identify additional studies. All pdf of full texts were saved in a dedicated folder shared between the authors in a private cloud. Duplicates were deleted. Study selection and collection lasted three months and the search was updated at each revision phase (last update January, 28 2024).

### Data categorisation

Categorization was carried out by three authors (M.N., B.M., F.C.) assessing therapeutic interventions reported in the articles identified. Two independent authors involved in Physical and Rehabilitation Medicine (PRM) used their expertise and referred to recent guidelines and/or systematic reviews regarding the topic of cLBP re-educational techniques to divide treatment protocols into 11 categories: Therapeutic Exercise (TE), Adapted Physical Exercise (APE), Adaptive Training Exercise/Complementary Medicine (CM), Manual Therapy (MT), Physical Agent modalities (PA), Education, Cognitive Re-education (CR), Multidisciplinarity, Kinesiotaping (KT), Sham Therapy (ST), No Intervention. It is important to highlight that most of these categories (TE, APE, MT, PA, Education, CR, Multidisciplinarity, KT and ST) were considered as physiotherapeutic approaches performed by a physiotherapist. Physiotherapy “is services provided by physiotherapists to individuals and populations to develop, maintain and restore maximum movement and functional ability throughout the lifespan. The service is provided in circumstances where movement and function are threatened by ageing, injury, pain, diseases, disorders, conditions and/or environmental factors and with the understanding that functional movement is central to what it means to be healthy^36^. Instead, Adaptive Training Exercise/Complementary Medicine are usually performed by professionals different from the physiotherapist”. We decided to include the RCTs focused on these techniques because the results (in terms of improvement of the LBP) have been widely demonstrated in the published peer-reviewed literature. The first step was to consider interventions regarding exercise, which can be defined as "a series of specific movements with the aim of training or developing the body by a routine practice or as physical training to promote good physical health"^36^. Many different types of treatments can fall under the term *exercise therapy (ET)*, each with its own design, duration, frequency, intensity, and mode of delivery. ET aims to increase muscle strength and function, to improve joint range of motion, and consequently reduce pain and increase mobility^29^. A key distinction has to be made between TE and APE. The former involves movement prescribed to correct impairments, restore muscular and skeletal function, and/or maintain a state of well-being, while APE involves exercise adaptations that could facilitate physical activity across a wide range of disabling conditions^37^. When LBP is caused by suboptimal postures that place excessive or damaging loads upon the spine APE is applied through postural techniques such as McKenzie, Souchard, or Pilates. In addition, active and passive movements can be differentiated according to the degree of activity expressed by the patient in performing the exercise. Another distinction involved MT: spinal manipulation differs from mobilisation because it is performed through the application of high-velocity impulses and thrusts administered beyond the normal joints’ range of motion (ROM), sometimes producing audible sounds. Physical agents are sources of energy that can be applied on the body surface with therapeutic purposes to improve the quality of life of the patient. They include heat, electrical current, vibration, laser, and ultrasounds, all of which are widely used for the treatment of chronic low back pain^38^. Various techniques derived from Eastern Medicine, such as Shiatsu, Tai-Chi, Qi Gong, and Yoga have been included in the *Complementary Medicine* category. The educational category consists of studies in which the main techniques were advice to the patients and the *Back School,* a technique developed in Sweden in 1969 consisting of patient education and exercises aimed at optimizing functional recovery. Another category became necessary for CR, a technique widely used in neurological disorders; CR can be effectively applied to cLBP to help patients become more aware of their condition and their pain, improve confidence to engage with normal activities of daily living, and reach their life goals and ultimately engage in a healthy lifestyle^39^. A final category involving a purely re-educational intervention is that regarding KT, a technique that uses of a thin functional elastic bandage applied to the patient's skin with the goal to reduce pain and increase blood flow and muscle performance while reducing muscle stiffness^40^. *Multidisciplinarity* was used when two or more techniques were used at the same time without one of them being predominant. Lastly, two more self-explanatory categories were needed to completely divide screened papers: Sham Therapy (ST) and No Intervention.

### Data items

Two authors (A.K., L.S.) independently performed data extraction. The following data at baseline were extracted: author and year of publication, journal of publication, men:women ratio, number of patients included with related mean age and BMI (kg/m^2^), mean length of symptoms duration prior to the physiotherapy, and the length of the follow-up. Data concerning the following patient-reported outcome measures (PROMs) were collected at baseline and at last follow-up: Visual Analog Scale (VAS) or numeric rating scale (NRS), Roland Morris Disability Questionnaire (RMQ)^41^ and Oswestry Disability Index (ODI)^42^. As VAS and NRS showed a high correlation, these were used interchangeably for the purpose of the present work^43^. Data were extracted in Microsoft Office Excel version 16.72 (Microsoft Corporation, Redmond, USA).

### Assessment of the risk of *bias* and quality of the recommendations

The risk of bias was evaluated in accordance with the guidelines in the Cochrane Handbook for Systematic Reviews of Interventions^44^. Two reviewers (A.K. and L.S.) evaluated the risk of bias in the extracted studies independently. Disagreements were solved by a third senior author (N.M.). RCTs were evaluated using the risk of bias of the software Review Manager 5.3 (The Nordic Cochrane Collaboration, Copenhagen). The following endpoints were evaluated: selection, detection, performance, attrition, reporting, and other biases.

### Synthesis methods

The statistical analyses were performed by the main author (F.M.) following the recommendations of the Cochrane Handbook for Systematic Reviews of Interventions^45^. Cohen’s Kappa (K) was used to quantify the inter-rater agreement among authors for full-text selection. The IBM SPSS version 25 was used. Cohen’s K was interpreted according to Altman’s definition^46^: K <0.2: poor, 0.2< K <0.4: fair, 0.41< K <0.60: moderate, 0.61< K <0.80: good, and K >0.81 excellent. For descriptive statistics, IBM SPSS version 25 was used. The mean and standard deviation were used. To assess baseline comparability, data distribution was analysed using the Shapiro-Wilk test. Analysis of variance (ANOVA) and the Kruskal-Wallis test were used for parametric and non-parametric data, with P values > 0.1 considered satisfactory. The network meta-analyses were performed using STATA SoftwareMP (version 14; StataCorporation, College Station, Texas, USA). The network meta-analyses were performed through the STATA routine for Bayesian hierarchical random-effects model analysis using the inverse variance method. The standardized mean difference (STD) was used for continuous data. The overall inconsistency was evaluated through the equation for global linearity via the Wald test. If P_Wald_ > 0.1, the null hypothesis could not be rejected, and the consistency assumption is accepted at the overall level of each treatment. Both confidence (CI) and percentile (PrI) intervals were set at 95% in each interval plot. Edge plots were performed to display direct and indirect comparisons and respective statistical weights. Interval plots were performed to rank treatments according to their estimated effect size. The funnel plots were performed to investigate the risk of bias related to each comparison. Greater plot asymmetries are associated with greater data variability, which indicates a greater risk of bias.

### Ethical approval

This study complies with ethical standards.

## Results

### Study selection

**Figure 1:**
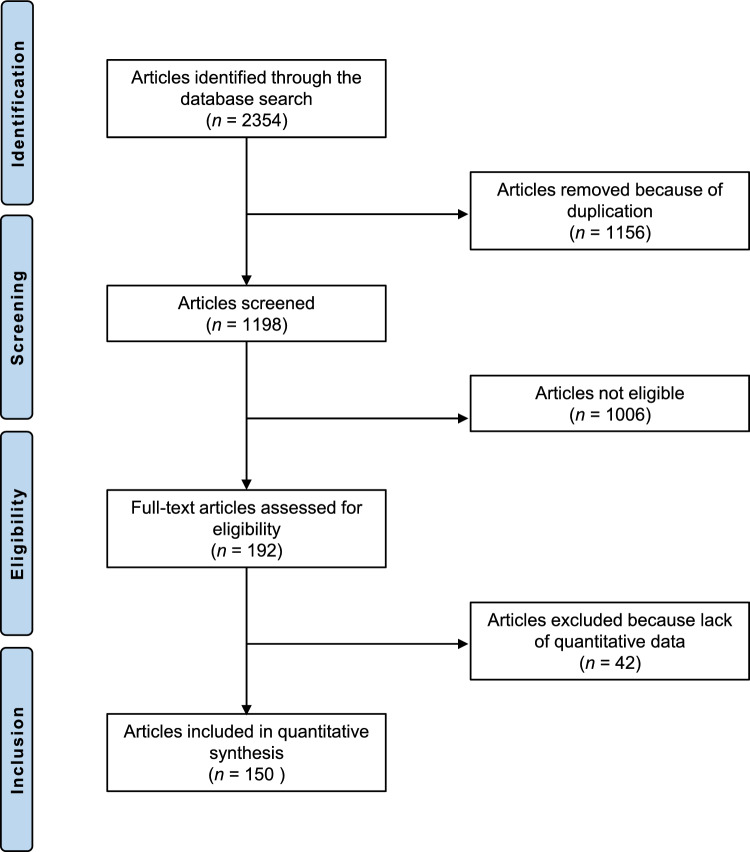
PRISMA flow chart of the literature search.

2354 RCTs were retrieved. A total of 1156 studies were excluded because they were duplicates. Another 1006 articles did not fulfil the eligibility criteria and were therefore discarded. Reasons for non-inclusion include in detail: study design (*N* = 697), low level of evidence (*N* = 148), therapy protocols that could not be classified into one of the 11 therapeutic categories of interest (TE, APE, CM, MT, PA, CR, KT, ST, Education, Multidisciplinarity, or No Intervention) (*N* = 149), and language limitations (*N* = 12). After full-text evaluation, an additional 42 investigations were excluded because quantitative data on the outcomes of interest were not available. Finally, 150 RCTs were available for inclusion. The inter-examiner agreement between the authors was good (Cohen's K = 0.71) for full-text selection. The results of the literature search are shown in Figure 1.

### Risk of *bias* assessment

**Figure 2:**
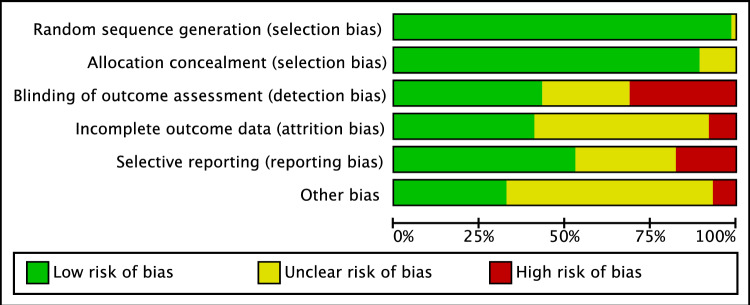
Cochrane risk of bias tool.

The analysis of the risk of bias showed a low risk of selection bias because all included studies were RCTs. The allocation of patients to each treatment group was performed with a high degree of quality in most studies, resulting in a low to moderate risk of allocation bias. Moderate risk was present for the risk of detection and performance bias, which was attributed to the lack of information on the blinding of investigators and patients during treatment and follow-up. In some studies, information on study dropouts during study enrollment or analysis was incompletely reported, resulting in moderate attrition bias. The risk of reporting bias was found to be overwhelmingly moderate, and the risk of other biases was mostly low. In summary, the risk of bias graph indicates a moderate quality methodological assessment of RCTs (Figure 2).

### Study characteristics and results of individual studies

Data from 12,773 patients were collected. The mean symptom duration was 61.2 ± 51.0 months and the mean follow-up was 4.3 ± 5.9 months. The mean age was 44.5 ± 9.4 years. The mean BMI was 25.8 ± 2.9 kg/m^2^. The generalities and demographics of the included studies are shown in Table 1.

**Table 1:** Generalities and patient baseline of the included studies.

Author, year	Journal	Class of treatment	Type of movement	Type of Treatment	Patients (n)	Follow-up (months)	Mean age	Women (%)
Aasa et al., 2015^47^	*J Orthop Sports Phys Ther*	Exercise	Low-load	Low-load motor control exercise	25	12	42.0	54
		Exercise	High-load lifting	High-load lifting exercise	28		42.0	57
Balthazard et al., 2012^48^	*BMC Musculoskelet Disord*	Spinal manipulation	High velocity, low amplitude	Spinal manipulation and active exercise (mobility, passive stretching, motor control, strenghtening)	19	6	44.0	36
		Physical agents	Ultrasound	Detuned US & active exercise (mobility, passive stretching, motor control, strenghtening)	18		42.0	30
Bhadauria et al., 2017^49^	*J Exerc Rehabil*	Exercise	Stabilization	Stabilization with verbal cues and tactile facilitation	12	0	32.8	50
		Exercise	Strenghtening	Dynamic strenghtening	12		36.7	42
		Pilates	Contraction	Isometric contraction	12		35.3	8
Cecchi et al., 2010^50^	*Clin Rehabil*	Back school	Individualized	Back school	68	12	57.9	70
		Physiotherapy	Individualized	Mobilization, active exercise, massage treatment of the soft tissues, proprioceptive neuromuscular facilitation	68		60.5	61
		Spinal manipulation	Mobilization, manipulation	Spinal manipulation	69		58.1	69
Costa et al., 2009^51^	*Phys Ther*	Motor control exercise	Individualized	Motor control exercise	77	10	54.6	58
		Sham		Detuned US and detuned short-wave therapy	77		52.8	62
Vibe Fersum et al., 2019^52^	*Eur J Pain*	Spinal manipulation	Individualized	Joint mobilization or manipulation (spine and pelvis)	59	36	43.1	52
		Cognitive functional therapy		Cognitive-behavioral	62		42.9	53
Garcia et al., 2013^53^	*Phys Ther*	Back school	Unknown	Back school	74	6	54.2	69
		Mckenzie	Symptom guided	Educational component and postural training (mckenzie)	74		53.7	78
Goldby et al., 2006^54^	*Spine*	Spinal stabilization	Stabilization	Spinal Stabilization & back school	35	24	43.4	68
		Spinal manipulation	Individualized	Spinal manipulation & back school	37		41.0	70
		Control		Back school	19		41.5	68
Halliday et al., 2016^55^	*J Orthop Sports Phys Ther*	Mckenzie	Symptom guided	Postural training (mckenzie)	32	2	48.8	80
		Motor control exercise	Contraction	Motor control exercise	30		48.3	80
Hohmann et al., 2018^56^	*Dtsch Arztebl Int*	Hirudotherapy		Hirudotherapy & back school	25	2	59.3	88
		Exercise	Various	Exercise & back school	19	1	56.5	95
Kääpä et al., 2006^57^	*Spine*	Multidisciplinary	Various	Group multidisciplinary rehabilitation: cognitive-behavioral stress management and applied relaxation sessions, back school education including occupational intervention and physical exercise program	59	24	46.0	98
		Physiotherapy	Various	Individual physiotherapy: light active exercise (muscle stretching, spine mobilization, and deep trunk muscle exercises) and passive treatment (massage, spine traction, spinal mobilization and TNSUS)	61		46.5	
Kobayashi et al., 2019^58^	*Complement Ther Med*	Shiatsu	Various	Shiatsu & standard care (compress or oral medicine)	30	2	67.4	67
		Standard care		Standard care (compress or oral medicine)	29		68.3	62
Lawand et al., 2015^59^	*Joint Bone Spine*	Exercise	Stretching	Global postural reeducation (Souchard)	30	6	49.4	81
		Control		Drugs	30		47.5	73
Macedo et al., 2019^60^	*Physiotherapy*	Kinesio taping with tension	Traction	Kinesiotaping	27	0	25.0	100
		Kinesio taping no tension		Kinesiotaping	27		24.0	
		Sham		Sham tape	27		25.0	
		Control			27		24.0	
Majchrzycki et al., 2014^61^	*Sci World J*	Massage	Deep tissue massage	Deep tissue massage	28	0	52.6	46
		Massage	Deep tissue massage	Dtm & nsaid	26		50.8	50
Murtezani et al., 2015^62^	*J Back Musculoskelet Rehabil*	Mckenzie	Symptom guided	Mckenzie	110	3	48.8	25
		Physical agents	Various	Interferential current, US, and heat	109		47.5	62
Sahin et al., 2018^63^	*Turk J Phys Med Rehab*	Physical agents	Various	PT (hot pack,US and TENS treatment) & exercise (strenghtening and stretching)	50	12	50.4	64
		Control	Stretching and strenghtening	Active isotonic and isometric strengthening exercises, stretching exercises	50		46.2	62
Saper et al., 2017^64^	*Ann Intern Med*	Yoga	Various	Yoga	127	9	46.4	57
		Aerobics	Various	Exercise	129		46.4	70
		Education		Education	64		44.2	66
Suh et al., 2019^65^	*Med*	Stretching	Stretching	Stretching	13	2	53.5	62
		Walking exercise	Walking	Walking exercise (WE)	13		54.2	85
		Spinal stabilization	Stabilization	Spinal stabilization	10		57.4	60
		Spinal stabilization	Stabilization, walking	Stabilization & WE	12		54.8	67
Takahashi et al., 2017^66^	*Fukushima J Med Sci*	Control		Nsaids	15	0	53.3	53
		Exercise	Stretching and strenghtening	Strengthening and stretching	18		57.6	56
Uzunkulaoğlu et al., 2018^67^	*Turk J Phys Med Rehabil*	Kinesio taping with tension	Traction	Kinesiotaping with tension	30	6	21.6	63
		Kinesio taping without tension		KT without tension	30		21.3	63
Yeung et al., 2003^68^	*J Altern Complement Med*	Exercise	Various	Warm up and stretching exercises	26	3	55.6	81
		Exercise	Various	Exercises & electroacupunture	26		50.4	85
Dufour et al., 2010^69^	*Spine*	Exercise	Strenghtening	Aerobic training and strengthening exercises & education	129	24	41.2	57
		Exercise	Strengthening	Strengthening exercises (intensive muscle training)	143		40.6	56
Helmhout et al., 2004^70^	*Spine*	Exercise	High-intensity strengthening	Progressive resistance muscle training	41	9	41.0	0
		Exercise	Low-intensity strengthening	Non-progressive, low-intensity resistance training	40		40.0	
Jarzem et al., 2005^71^	*J Musculoskelet Pain*	Sham		Sham	83	1	45.1	50
		Tens		Tens	84			
		Acupuncture TENS		Acupuncture TENS	78			
		Tens	Biphasic	Tens	79			
Meng et al., 2011^72^	*Clin J Pain*	Back school		Back school	181	12	50.2	65
		Back school		Back school	163		49.5	63
Prommanon et al., 2015^73^	*J Phys Ther Sci*	Back care pillow		US, hot pack and back care pillow	26	3	38.5	42
		Control		US and hot pack	26		39.7	50
Tavafian et al., 2011^74^	*Clin J Pain*	Education		Education & drugs	97	6	44.6	73
		Control		Control & drugs	100		45.9	83
Tavafian et al., 2014^75^	*Int J Rheum Dis*	Education		Education & drugs	87	12	44.6	75
		Control		Control & drugs	91		46.2	82
Alfuth et al., 2016^76^	*Orthopäde*	Mobilization	Mobilization	Mobilization	14	1	50.0	79
		Stabilization	Stabilization	Stabilization	13		43.0	54
Grande-Alonso et al., 2019^77^	*Pain Med*	Multidisciplinary	Various	Multidisciplinary	25	3	39.9	56
		Multidisciplinary	Stabilization	Multidisciplinary	25		38.3	56
Ali et al., 2019^78^	*J Bodyw Mov Ther*	Spinal manipulation	Various	Spinal manipulation	14	0	35.4	
		Spinal manipulation	Various	Spinal manipulation	14		35.3	
Ahmadi et al., 2020^79^	*Clin Rehabil*	Exercise	Individualized	Exercise (Feldenkrais method) & education	30	0	42.6	100
		Education	Individualized	Home-based exercise & education	29		38.9	100
Almhdawi et al., 2020^80^	*Clin Rehabil*	Exercise	Strengthening, stretching	Strengthening and stretching exercises	21	0	40.5	34
		Control			20		41.7	20
Added et al., 2016^81^	*J. Orthop. Sports Phys. Ther.*	Physiotherapy	Individualized	Manual therapyexercises	74	6	44.6	72
		Physiotherapy	Individualized	Exercises & KT	74	6	45.6	72
Arampatzis et al., 2017^82^	*Eur J Appl Physiol*	Exercise	Lowmoderate intensity	Lowmoderate intensity exercises	20	0	31.9	40
		Control			20		31.4	45
Areeudomwong et al., 2016^83^	*Musculoskeletal Care*	Exercise	Contraction	Proprioceptive neuromuscular facilitation & contraction exercises	21	3	35.4	71
		Control			21		36.2	76
Bae et al., 2018^84^	*J Back Musculoskelet Rehabil*	Exercise	Core stabilization	Core stabilization	18	3	32.7	50
		Exercise	Strenghtening	Strenghtening exercises	18		32.4	61
Bi et al., 2013^85^	*Int J Med Res*	Exercise	Contraction	Pelvic floor muscle exercise	23	0	29.1	44
		Control	Strenghtening	US, short-wave diathermy and strengthening exercises	24		30.9	46
Bicalho et al., 2010^86^	*Man Ther*	Manipulation	High-velocity	Lumbar manipulation	20	0	29.5	75
		Control			20		26.5	60
Bronfort et al., 2011^87^	*Spine J*	Exercise	Various	Education & simple exercises	101	9	45.6	58
		Spinal manipulation	High-velocity, low-amplitude	Spinal manipulation	100		45.2	66
		Exercise	Strengthening	Strenghtening exercises	100		44.5	57
Cai et al., 2017^88^	*Med Sci Sports Exerc*	Exercise	Resistance	Resistance exercises	25	4	28.9	50
		Exercise	Contraction	Isometric contraction	24		26.1	
		Exercise	Stabilization	Stabilization	25		26.9	
Azevedo et al., 2017^89^	*Phys Ther*	Exercise	Strengthening, stretching	Strengthening and stretching exercises	74	4	40.4	58
		Control	Various	Education & exercises	74		43.4	65
Castro-Sánchez et al., 2016^90^	*Spine J*	Spinal manipulation	High-velocity	Manipulation	31	0	43.0	65
		Spinal manipulation	Low-velocity	Manipulation	31		47.0	61
Ryan et al., 2010^91^	*Man Ther*	Exercise	Various	Aerobic exercises	20	3	45.2	70
		Control		Education	18		45.5	61
Chhabra et al., 2018^92^	*Eur Spine J*	App	Various	Exercises	45	0	41.4	
		Control		Drugs	48		41.0	
Cortell-Tormo et al., 2018^93^	*J Back Musculoskelet Rehabil*	Exercise	Various	Exercises for coordinated contraction of transversus abdominis with lumbar multifidus	11	0	35.6	100
		Control		No intervention	8		35.6	100
Cruz-Díaz et al., 2015^94^	*Disabil Rehabil*	Pilates	Individualized	Strengthening exercises	53	11	69.6	100
		Physiotherapy	Various	Tens	48		72.7	100
Cruz-Díaz et al., 2017^95^	*Complement Ther Med*	Pilates	Various	Pilates	34	0	36.9	68
		Pilates	Various	Pilates	34		35.5	62
		Control		No intervention	30		36.3	63
Cuesta-Vargas et al., 2011^96^	*Am J Phys Med Rehabil*	Multidisciplinary	Various	Multimodal rehab	24	0	37.6	58
		Multidisciplinary	Various	Multimodal rehab	25		39.8	54
Diab et al., 2013^97^	*J Back Musculoskelet Rehabil*	Exercise	Traction	Lumbar traction	40	6	46.3	45
		Exercise	Stretching	Strenghtening exercises	40		45.9	43
Koldaş Doğan et al., 2008^98^	*Clin Rheumatol*	Aerobics	Walking	Aerobic exercises	19	1	37.1	79
		Physical agents	Various	Hot packs & US & TENS	18		41.5	78
		Control		Mobilitazion and stretching	18		42.1	78
Eardley et al., 2013^99^	*Forsch Komplementmed*	Multidisciplinary	Individualized	Therapeutic conversation	20	0	48.8	85
		Sham	Individualized	Sham exercises	21		48.1	67
		Delayed			17		44.6	65
Engbert et al., 2011^100^	*Spine*	Exercise	Climbing	Climbing exercises	10	0	51.9	60
		Exercise	Various	Trunk stabilization and strenghtening	13		50.4	46
de Oliveira et al., 2013^101^	*Phys Ther*	Spinal manipulation	High-velocity	Spinal manipulation	74	0	46.0	68
		Spinal manipulation	Region-specific	Spinal manipulation	74		46.3	80
França et al., 2012^102^	*J Manipulative Physiol Ther*	Exercise	Stabilization	Spinal stabilising exercises	15	0	42.1	
		Exercise	Stretching	Strenghtening exercises	15		41.5	
Friedrich et al., 1998^103^	*Arch Phys Med Rehabil*	Exercise	Various	Motivational intervention	44	12	43.3	57
		Exercise	Various	Spinal mobility & trunk and lower limbs exercises	49		44.9	45
Frost et al., 1995^104^	*BMJ*	Exercise	Aerobics	Stretching & aerobic exercises	36	6	34.2	53
		Control		Back school	35		38.5	51
Garcia et al., 2017^105^	*BMJ*	Mckenzie	Various	Mckenzie	74	11	57.5	78
		Control		Detuned pulsed ultrasound	73		55.5	74
Gardner et al., 2019^106^	*BMJ*	Exercise	Individualized	Individualized exercises	37	10	44.0	66
		Control	Various	Exercise	38		45.0	49
Gavish et al., 2015^107^	*Physiotherapy*	Exercise	Oscillation	Mobilization	18	1	53.2	33
		Control		No intervention	18		47.1	56
Geisser et al., 2005^108^	*Clin J Pain*	Exercise	Various	Self-corrections & stretches & strengthening exercises	21	0	39.3	67
		Exercise	Various	Exercises & sham manipulation	18		38.7	56
		Non-specific exercise	Various	Non-specific exercise	15		36.5	80
		Non-specific exercise	Various	Non-specific exercise & sham manipulation	18		46.3	61
Gwon et al., 2020^109^	*Physiother Theory Pract*	Exercise	Side bridge	Vibration & side-lying bridge exercise on a sling suspension system	15	0	21.9	2
		Exercise	Side bridge	Side-lying bridge exercise on a sling suspension system	15		21.6	2
Haas et al., 2014^110^	*Spine J*	Sham	Various	Sham manipulation	95	11	40.9	49
		Spinal manipulation	High velocity, low amplitude	Spinal manipulation	99		41.4	49
		Spinal manipulation	High velocity, low amplitude	Spinal manipulation	97		41.8	49
		Spinal manipulation	High velocity, low amplitude	Spinal manipulation	100		41.2	52
Halliday et al., 2019^111^	*Physiotherapy*	Mckenzie	Symptom guided	Mckenzie	35	10	48.8	80
		Motor control exercise	Contraction	Motor control exercises	35		48.3	80
Harts et al., 2008^112^	*Aust J Physiother*	Exercise	High intensity	High-intensity progressive resistance exercise	23	4	44.0	0
		Exercise	Low intensity	Low-intensity resistance exercise program	21		42.0	
		Control		No intervention	21		41.0	
Macedo et al., 2015^113^	*Phys Ther*	Exercise	Various	Individualized and submaximal exercises	86	12	49.6	52
		Motor control exercise	Symptom guided	Spine and pelvis stabilization	86		48.7	66
Javadian et al., 2012^114^	*J Back Musculoskelet Rehabil*	Exercise	Stabilization	Spinal stabilization	30	3		
		Exercise	Various	Active mobilization exercises				
Loss et al., 2020^115^	*Chiropr Man Ther*	Spinal manipulation	Thrust	Lumbar manipulation	12	0	41.7	50
		Control	Various	Sham manipulation	12		43.9	50
Kell et al., 2011^116^	*J Strength Cond Res*	Exercise	Strengthening	Strengthening exercises	60	0	42.4	31
		Exercise	Strengthening	Strenghtening exercises	60		41.7	37
		Exercise	Strengthening	Strenghtening exercises	60		42.8	33
		Control		No intervention	60		43.2	38
Kim et al., 2015^117^	*Clin Rehabil*	Exercise	Contraction	Core exercises & TENS	27	2	29.7	100
		Control		Tens	26		28.6	
Kim et al., 2018^118^	*J Sport Rehabil*	Exercise	Various	Exercises Using a sling	38	3	39.5	61
		Exercise	Stabilization	Stabilizing exercise	39		46.2	54
Tekur et al., 2008^119^	*J Altern Complement Med*	Yoga	Various	Yoga	40	0	49.0	53
		Exercise	Various	Active mobilization exercises	40		48.0	38
Tekur et al., 2012^120^	*Complement Ther Med*	Yoga	Various	Yoga	40	0	49.0	53
		Exercise	Various	Active mobilization exercises	40		48.0	38
de Oliveira et al., 2020^121^	*J Physiother*	Spinal manipulation	High-velocity	High-velocity thrust manipulation	71	5	45.0	77
		Spinal manipulation	Various	‘Generic manipulation’	72		45.0	78
Zou et al., 2019^122^	*Medicina*	Tai chi	Various	Tai chi	15	0	58.1	73
		Exercise	Stabilization	Bridge exercises	15		58.4	73
		Control		No intervention	13		60.7	77
Zhang et al., 2014^123^	*J Int Med Res*	Education	Strenghtening	Education sessions once a week for 12 weeks	25	0	22.3	33
		Control	Strenghtening	Lumbar strengthening exercises	24		23.0	41
Zheng et al., 2012^124^	*J Tradit Chin Med*	Massage	Pressure; traction	Deep massage to the tender point and peripheral taut band	30	0	43.0	44
		Control	Traction	Sham manipulation	30		42.0	50
Yang et al., 2021^125^	*J Bodyw Mov Ther*	Pilates	Various	Pilates	20	5	50.5	75
		Control		Education program regarding low back pain	19		47.9	79
Waseem et al., 2018^126^	*J Back Musculoskelet Rehabil*	Exercise	Core stabilization	Core & pelvic floor exercises	53	0	46.4	34
		Exercise	Various	Stretching	55		45.5	35
Williams et al., 2005^127^	*Pain*	Yoga	Various	Yoga	20	0	48.7	65
		Control		Two 1-h lectures on occupationalphysical therapy	24		48.0	71
Verbrugghe et al., 2021^128^	*Int J Environ Res Public Health*	Exercise	High-intensity strengthening	Cardiorespiratory training, general resistance training, and core muscle training - high intensity	16	6	44.3	68
		Control	Moderate-intensity strengthening	Cardiorespiratory training, general resistance training, and core muscle training - moderate intensity	13		44.0	68
Sipaviciene et al., 2020^129^	*Clin Biomech*	Exercise	Stabilization	Dynamic stretching exercises & lumbar stabilization	35	3	38.3	100
		Exercise	Strengthening	Lumbar muscle strengthening exercise	35		38.5	100
Phattharasupharerk et al., 2018^130^	*J Bodyw Mov Ther*	Qi gong	Various	Qi gong	36	0	35.7	67
		Control		General advice on managing low back pain	36		34.8	61
Magalhães et al., 2018^131^	*Braz J Phys Ther*	Exercise	Various	Stretching exercises of main muscle groups and motor control exercises	33	0	46.6	76
		Exercise	Various	Progressive and sub-maximal exercises	33		47.2	73
Monticone et al., 2013^132^	*Clin J Pain*	Exercise	Various	Cognitive-behavioral	45	12	49.0	60
		Control	Various	Active and passive mobilizations of the spine, and exercises aimed at stretching and strengthening muscles, and improving postural control	45		49.7	56
Monticone et al., 2014^133^	*Eur Spine J*	Motor control exercise	Stabilizing	Spinal stabilising exercises	10	3	58.9	70
		Control	Various	Passive spinal mobilisation, stretching, muscle strengthening, and postural control	10		56.6	40
Morone et al., 2011^134^	*Eur J Phys Rehabil Med*	Back school	Various	Theoretical lessons	41	5	61.2	59
		Control		Drugs	29		58.6	72
Matarán-Peñarrocha et al., 2020^135^	*Clin Rehabil*	Exercise	Various	Supervised exercise programme	32	6	54.3	53
		Exercise		Non-supervised home exercise programme	32		53.2	47
Laosee et al., 2020^136^	*Complement Ther Med*	Massage	Pressure	Traditional thai massage	70	3	68.2	77
		Massage	Pressure	Traditional thai massage	70		69.1	71
Rittweger et al., 2002^137^	*Spine*	Exercise	Extention	Isodynamic lumbar extension exercise	25	6	49.8	44
		Exercise	Vibration	Wbv	25		54.1	52
Prado et al., 2019^138^	*Physiother Theory Pract*	Exercise	Stretching	Active exercises	27	0	35.0	70
		Control		No intervention	27		33.0	63
Vollenbroek-Hutten et al., 2004^139^	*Clin Rehabil*	Multidisciplinary	Various	Multimodal rehab	69	6	38.5	
		Control		No intervention	73		39.5	
del Pozo-Cruz et al., 2011^140^	*J Rehabil Med*	Exercise	Vibration	Wbv	25	0	58.7	74
		Control		No intervention	24		59.5	72
Kostadinovic et al., 2020^141^	*J Back Musculoskelet Rehabil*	Exercise	Stabilization; mobilization	Lumbar stabilization exercises and thoracic mobilization & TENS	40	0	44.1	55
		Exercise	Stabilization	Lumbar stabilization & TENS	40		44.3	58
Monticone et al., 2015^142^	*Eur J Pain*	Exercise	Individualized	Exercises	75	24	53.2	63
		Control		Ergonomic advice	75		53.8	60
Járomi et al., 2018^143^	*J Clin Nurs*	Back school	Various	Isometric & isotonic exercises	67	0	41.7	94
		Control		Brief writen lifestyle guidance	70		41.1	93
Liu et al., 2019^144^	*Int J Environ Res Public Health*	Tai chi	Various	Tai chi	15	0	58.1	73
		Exercise	Stabilization	Active exercises (bridge)	15		58.4	73
		Control		No intervention	13		60.7	77
Lara-Palomo et al., 2012^145^	*Clin Rehabil*	Massage	Interferential current	Interferential current	30	0	50.0	70
		Massage	Superficial pressure	Superficial manual massage	31		47.0	65
Saha et al., 2019^146^	*Complement Ther Clin Pract*	Massage	Pressure	Spinal manipulation	25	1	52.2	68
		Control		No intervention	25		47.2	88
Segal-Snir et al., 2016^147^	*J Back Musculoskelet Rehabil*	Exercise	Rotation	Rotation exercises	20	1	57.2	100
		Control		No intervention	15		54.7	100
Nambi et al., 2014^148^	*Int J Yoga*	Yoga	Various	Yoga	30	6	44.3	63
		Control	Strenghtening, stretching	Strengthening and stretching of the abdominal and back muscles	30		43.7	43
Salamat et al., 2017^149^	*J Bodyw Mov Ther*	Exercise	Stabilization	Stabilization exercise	12	0	35.8	
		Motor control exercise	Various	Exercises training to modify pain provocative postures and movement patterns	12		36.1	
Salavati et al., 2015^150^	*J Bodyw Mov Ther*	Exercise	Stabilization	Routine physiotherapy plus supervised, intensive stabilizing exercises	20	0	32.6	0
		Control	Various	Interferential therapy	20		29.9	0
Masharawi et al., 2013^151^	*J Back Musculoskelet Rehabil*	Exercise	Various	Exercises	20	2	52.5	100
		Control		No intervention	20		53.6	100
Natour et al., 2014^152^	*Clin Rehabil*	Pilates	Various	Pilates	30	3	47.8	80
		Control		No intervention	30		48.1	77
Murtezani et al., 2011^153^	*Eur J Phys Rehabil Med*	Exercise	Individualized	High-intensity aerobis exercise	50	0	51.4	48
		Control	Various	IFC, TENS, ultrasound, heat	51			49
Kogure et al., 2015^154^	*PLoS One*	Spinal manipulation	Various	Arthrokinematic Approach-Hakata method	90	6	60.0	60
		Sham	Various	Sham manipulation	89		59.6	64
Ozsoy et al., 2019^155^	*Dove Med Press*	Exercise	Core stabilization	Core stability training	21	0	68.1	29
		Exercise	Various	Core stability exercise and myofascial release technique	21		68.0	31
Jousset et al., 2004^156^	*Spine*	Multidisciplinary	Various	Multimodal rehab	43	5	41.4	30
		Exercise	Individualized	Active exercises	41		39.4	37
Gracia et al., 2013^53^	*Phys Ther*	Back school	Various	Back school	74	5	54.2	69
		Mckenzie	Individualized	Mckenzie	74		53.7	78
Roche-Leboucher et al., 2011^157^	*Spine*	Exercise	Various	Isotonic exercises	68	12	40.8	32
		Exercise	Various	Isotonic exercises	64		38.7	38
Khalil et al., 1992^158^	*Spine*	Spinal manipulation	Stretching	Spinal manipulation	14	0	41.1	43
		Control		Multimodal rehab	14		48.5	50
Mannion et al., 1999^159^	*Spine*	Exercise	Various	Isometric exercises	46	6	46.3	61
		Aerobics	Low-impact	Stretching and aerobic and muscle-toning exercises	47		45.2	54
		Physical agents	Various	Variuous physical agents	44		43.7	55
Mannion et al., 2001^160^	*Spine*	Exercise	Various	Strengthening, coordination and aerobic exercises	44	12	46.3	61
		Aerobics	Low-impact	Stretching and aerobic and muscle-toning exercises	43		45.2	54
		Physical agents	Various	Physical therapy	40		43.7	55
Yoon et al., 2012^161^	*Ann Rehabil Med*	Massage	Symptom guided	Deep cross-friction massge	12	1	50.3	58
		Tens	Various	Tens	10		53.3	60
Yang et al., 2019^162^	*J Healthc Eng*	Exercise	Symptom guided	Exercises	5	0	35.0	20
		Control	Various	Manual therapy	3		50.3	100
Hicks et al., 2016^163^	*Clin J Pain*	Control	Various	Moist heat treatment & US	31	3	69.5	52
		Exercise	Stabilization	Trunk muscle training program augmented with neuromuscular electrical stimulation	26		70.7	58
Yalfani et al., 2020^164^	*J Bodyw Mov Ther*	Water pilates	Various	Water pilates	12	0	25.2	100
		Pilates	Various	Pilates	12		24.7	100
Trapp et al., 2015^165^	*J Back Musculoskelet Rehabil*	Exercise	Feedback	Exercises with biofeedback	15	0	45.5	33
		Control	Various	Exercises & walking	15		40.6	40
Kofotolis et al., 2016^166^	*J Back Musculoskelet Rehabil*	Pilates	Various	Pilates	37	0	42.7	100
		Control		No intervention	28		41.2	100
		Exercise	Strengthening	Trunk strenghtening exercises	36		39.1	100
Kuvacic et al., 2018^167^	*Complement Ther Clin Pract*	Control		Education	15	0	33.6	53
		Yoga	Various	Yoga	15		34.7	40
Hernandez-Reif et al., 2001^168^	*Intern J Neuroscience*	Massage	Various	Manual therapy	24	0	43.8	58
		Control	Various	Muscle relaxation exercise			36.7	50
Lewis et al., 2005^169^	*Spine*	Exercise	Various	Active aerobic exercises	33	12	46.1	65
		Exercise	Individualized	Manual therapy	29		45.7	65
O’Keeffe et al., 2020^170^	*J Sports Med*	Cognitive functional therapy	Individualized	Cognitive functional therapy	106	10 to 10.5	47.0	77
		Exercise	Various	Exercises & relaxation & pain education	100		50.6	70
Kaeding et al., 2017^171^	*Scand J Med Sci Sports*	Exercise	Vibration	Whole body vibration	21	0	46.4	67
		Control		No intervention	20		44.6	70
Petrozzi et al., 2019^172^	*Chiropr Man Therap*	App	Various	Cognitive-behavioral & exercises	54	< 10	50.1	54
		Control	Various	Manual therapy	54		50.6	59
Winter et al., 2015^173^	*J Back Musculoskelet Rehabil*	Exercise	Rotation	Hip muscles rotation-stetching	10	0	45.9	45
		Exercise	Stretching	Multi-directional hip stretching	10		48.9	
		Exercise	Strengthening	Strenghtening exercises	10		38.3	
Massé-Alarie et al., 2017^174^	*Clin Neurophysiol*	Physical agents	Contraction	Magnetic neurostimulation at lumbar level	11	0	33.2	45
		Sham	None	Sham magnetic stimulation	10		42.1	50
Martí-Salvador et al., 2018^175^	*Arch Phys Med Rehabil*	Spinal manipulation	Various	Manipulation	33	2	43.4	52
		Sham	Various	Sham spinal manipulation	33		41.7	61
Aguilar-Ferrándiz et al., 2022^176^	*Nature*	Kinesio taping	Without tension	KT application at paravertebral musculature	29	0	44.0	59
		Tens		Tens	29		46.0	72
Elgendy et al., 2022^177^	*Ortop Traumatol Rehabil*	Physical agents	Various	Shock waves	15	0	32.7	
		Control	Stretching, strengthening	Stretching & strenghtening exercises	15		33.3	
Fukuda et al., 2021^178^	*Braz J Phys Ther*	Spinal manipulation	Joint mobilzation	Manual therapy & lumbar stabilization	35	12	35.2	53
		Spinal manipulation	Joint mobilization, strengthening	Manual therapy & lumbar stabilization	35		40.2	
Ma et al., 2021^179^	*Ann Palliat Med*	Physical agents	Needling	Fu's subcutaneus needle	30	12	47.7	50
		Massage	Swedish massage	Swedish massage	30		49.2	63
Maggi et al., 2022^180^	*Aging Clin Exp Res*	Kinesio taping	No tension	KT application at lumbar spine	57	3	66.8	72
		Control		Back school	62		67.8	82
Jalalvandi et al., 2022^181^	*BMC Musculoskelet Disord*	Exercise	Stretching, strengthening	Exercises for strengthening and stretching of the back and pelvis muscles	22	0	37.9	73
		Tens		Tens	22		36.1	64
Atilgan et al., 2021^182^	*J Back Musculoskelet Rehabil*	Exercise	Breathing, stabilization	Breathing exercises & core stabilization exercises	23	0	32.1	100
		Control	Stabilization	Lumbar stabilization	20		37.7	100
Pivovarsky et al., 2021^183^	*Einstein (Sao Paulo)*	Sham		Sham TENS	35	0	40.8	69
		Tens		Tens	35		44.0	66
		Tens		Tens	35		42.6	77
Van Dillen et al., 2020^184^	*JAMA Neurol*	Exercise	Various	Active exercises training for lumbar spine	74	12	42.4	68
		Exercise	Stretching, strengthening	Strength and flexibility exercises	75		42.6	55
Vibe Fersum et al., 2012^185^	*Eur J Pain*	Spinal manipulation	Joint mobilization	Joint mobilization or manipulation	43	0	42.9	49
		Cognitive functional therapy	Unknown	Cognitive-functional therapy	51		41.0	53
Ghroubi et al., 2007^186^	*Ann Readapt Med Phys*	Spinal manipulation	Symptom guided	Spinal manipulation	32	1	39.1	84
		Sham		Sham spinal manipulation	32		37.4	75
Huber et al., 2019^187^	*BMC Musculoskelet Disord*	Walking	Walking	Guided hiking in mountains	27	14	52.9	52
		Walking	Walking, heat	Balneotherapy	26		53.4	54
		Control		No intervention	27		43.8	63
Werners et al., 1999^188^	*Spine*	Tens		Tens	74	3	38.3	43
		Massage	Traction	Lumbar traction	73		39.2	49
Kankaanpää et al., 1999^189^	*Spine*	Exercise	Various	Training of trunk muscles	30	12	39.8	37
		Control	Various	Manual therapy	24		39.3	33
Marshall et al., 2008^190^	*Spine*	Spinal manipulation	High velocity, low amplitude, various	Isometric then concentricexcentric exercises	12	9	34.3	50
		Spinal manipulation	High velocity, low amplitude	Manipulation	13		35.8	54
		Spinal manipulation	Non thrust, various	Abdominal stabilization	12		33.9	50
		Spinal manipulation	Non thrust	Education on how to stay active	13		41.7	42
Branchini et al., 2015^191^	*F1000research*	Spinal manipulation	Pressure	Manual therapy and fascial manipulation	11	3	48.0	64
		Control	Individualized	Respiratory reeducation, propioception & stretching & core stability exercises,	13		44.0	69
Batıbay et al., 2020^192^	*J Orthop Sci*	Pilates	Various	Pilates	28	0	49.3	100
		Exercise		Pelvic tilt, stretching and strenghtening exercises	25		48.4	100
Elabd et al., 2020^193^	*J Appl Biomech*	Exercise	Stabilization, stretching	Lumbar stabilization	25	0	26.8	52
		Exercise	Stabilization	Lumbar stabilization	25		27.4	
Dadarkhah et 2020^194^	*J Natl Med Assoc*	Exercise	Core stabilization	Flexibility & strengthening & cool-down exercises	28	12	49.0	57
		Exercise	Core stabilization	Flexibility & strengthening & cool-down exercises	28		50.0	57
Nardin et al., 2022^195^	*Lasers Med Sci*	Exercise	Aerobics	Deep water running	20	1	42.2	80
		Sham	Aerobics	Sham	20		42.8	75
		Physical agents		Laser-therapy (THOR DD2)	20		43.1	80

### Pain

**Figure 3:**
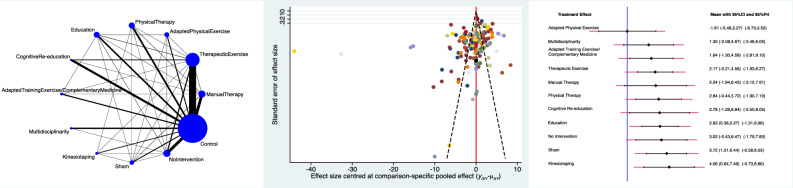
From left to right: edge, interval, and funnel plot of the comparison pain.

The Adapted Physical Exercise group evidenced the lowest pain score (SMD −1.61; 95% CI −5.48 to 2.27), followed by Multidisciplinary (SMD 1.30; 95% CI −2.08 to 4.67) and Adapted Training Exercise/Complementary Medicine (SMD 1.64; 95% CI −1.30 to 4.59). The equation for global linearity found no statistically significant inconsistency (P_Wald_ = 0.1). These results are shown in Figure 3.

#### RMQ

**Figure 4:**
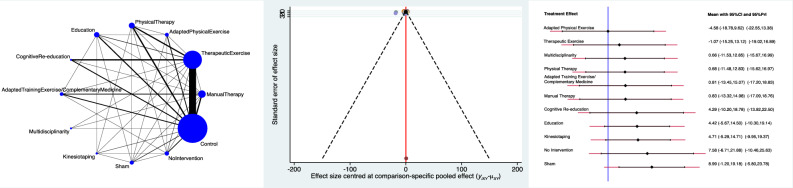
From left to right: edge, interval, and funnel plot of the comparison RMQ.

The Adapted Physical Exercise group evidenced the lowest RMQ score (SMD −4.58; 95% CI −18.78 to 9.62) followed by Therapeutic Exercises (SMD −1.07; 95% CI −15.25 to 13.12) and Multidisciplinary (SMD 0.66; 95% CI −11.53 to 12.85). The equation for global linearity found no statistically significant inconsistency (P_Wald_ = 0.2). These results are shown in Figure 4.

#### ODI

**Figure 5:**
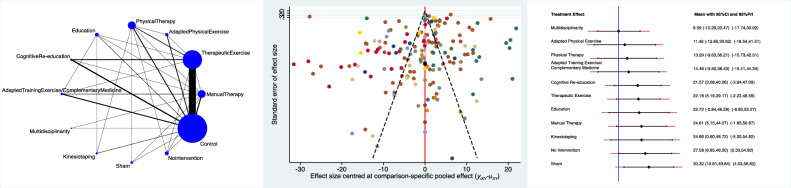
From left to right: edge, interval, and funnel plot of the comparison ODI.

The Multidisciplinary group evidenced the lowest ODI score (SMD 6.59; 95% CI −10.29 to 23.47), followed by Adapted Physical Exercise (SMD 11.49; 95% CI −12.65 to 35.62) and Physical Agent modalities (SMD 13.29; 95% CI −9.63 to 36.21). The equation for global linearity found no statistically significant inconsistency (P_Wald_ = 0.08). These results are shown in Figure 5.

## Discussion

Within the considered physiotherapeutic and non-conventional approaches to manage nonspecific and/or mechanic cLBP, adapted physical exercise, physical agent modalities, and a multidisciplinary approach seemt to represent the most effective strategy in reducing pain and disability.

One of the main difficulties in comparing different types of physiotherapeutic management in cLBP is the lack of a comprehensive and widely accepted classification of the various available therapies. The present work is based on a novel, expert-based classification of the different types of physiotherapeutic and non-conventional approaches available for the management of cLBP. While different classifications have been proposed over time, none has been able to successfully highlight the different effectiveness of each kind of management in terms of disability and pain levels^30,31^. As opposed to the previously published works, the presented classification was able not only to include all the treatments available in the current literature but also to differentiate between the efficacy of different types of management. Hopefully, this classification will simplify comparisons between different types of regimens.

APE showed to be one of the most efficient physiotherapeutic strategy, and it is also one of the most investigated commonly management option in the literature. The results of the present work contrast with those of a recent network meta-analysis (NMA) that compared different types of exercise and physiotherapeutic management in the setting of cLBP^196^. While there is agreement that PE and MT are less effective than active therapy options, Owen et al.^196^ reported no-to-low evidence for the efficacy of Pilates and McKenzie regimens for the management of cLBP. Both therapeutic options fall in the same APE category in the present work. This allowed to aggregate data from different studies and achieve a higher numerosity for the analysed category. In turn, this might have led to stronger evidence supporting APE in the present work. In support of the role of APE in the setting of cLBP, a recent NMA by Fernandez-Rodriguez et al.^28^ showed that the most effective treatment protocol included, among others, at least one session of Pilates or strength exercise per week. Similar results were also obtained by Hayden et al.^197^, who compared APE schemes to other exercise and treatment types, and concluded that Pilates and McKenzie regimens promoted functional restoration and reduced pain intensity.

Recently, APE has gained popularity for the management of cLBP, and its use has been supported by a number of publications^198–203^. In addition to its efficacy, APE presents further advantages such as the possibility of individualizing the therapeutic regimen according to the specific needs and interests of the patients^204,205^. These characteristics can increase compliance with the management^197^ and, consequently, its efficacy. Furthermore, APE protocols have been applied safely in elderly and fragile cLBP patients, a particularly relevant group considering population aging^205^. In this setting, APE seems to be able not only to improve pain and function but also to reduce the fear of falling and increase balance^205^. Interestingly, while improving symptoms and function, APE does not seem to increase trunk muscle size^55^. This finding might be related to the short duration of the study (eight weeks)^55^, but might also indicate that the efficacy of APE does not only rely on muscule size. This, in turn, might explain why APE was more effective than other forms of exercise. Possible intervening mechanisms might be the focus of APE on functional improvement or balance, or the encouraging effects of APE on psychosocial outcomes^206^ and improvement of kinesiophobia^207,208^: further studies will be required to understand more clearly why this type of management is particularly effective in patients with cLBP.

This important finding can be explained considering that active physiotherapy involves the active participation of the patient in performing therapeutic exercises or activities that promote mobility, strength, and functional improvement^17^. It encourages patients to actively participate in their rehabilitation, fostering self-management and independence^17^. This translates into a greater awareness of patients of their means, in adapting their body to the surrounding environment. Patient do not feel that they have a disability that limits the activities of daily living, but, thanks to the Adapted Physical Exercise, subjects develop the means to differently tackle the required tasks.

The application of physical agents also proved to be an effective strategy for the management of cLBP. Passive physiotherapy refers to interventions where the patient receives treatment without actively engaging in physical movements, as happens during the application of the physical agents. It relies on external therapeutic interventions facilitated by the physiotherapist on the affected muscles, which often appear hypercontracted in case of pain. Passive stretch reduces stiffness (viscoelastic stress relaxation) and decreases stretch-induced pain^16^. This could represent the first step to consequently work on the functional use of these muscles, as it happens in APE. In other terms, passive treatment can help with immediate pain relief, but active treatment keeps the patient functional in the long term.

Lastly, considering the weight of psychosocial factors in the setting of cLBP^209^, it is not surprising that multimodal therapy was effective under the outcomes of interest considered. Furthermore, the available evidence supports the hypothesis that multimodal management exerts a positive influence in return to work^210^ and reduction of work absenteeism^211^. Heitz et al.^212^ identified several modifiable and non-modifiable risk factors for the development of persistent cLBP in patients with subacute and cLBP, 56 of them somatic and 61 of them psychosocial. These figures show clearly that focussing solely on the somatic aspects leaves out a vast number of psychological factors involved in the development of cLBP. These data and the evidence presented in the present work thus support the inclusion of psychologic management in the therapy of nonspecific cLBP. While similar positive findings around the employment of multimodal management in cLBP have been reported by different studies^213–217^, future research should focus on what type of psychological therapy is best used in what type of setting^215^.

This work does not come without limitations. The main one is represented by the heterogeneity in the inclusion criteria and therapeutic schemes in the available literature. Future studies should focus on adopting a uniform classification of different therapeutic options to allow easier comparability, and larger cohorts with sub-analysis of patients in different age ranges or with different symptom durations will be helpful to analyze whether different patient cohorts can benefit from different management options. Three trained physical therapists (M.N., B.M., F.C.) collectively performed data categorisation to reduce the risk of bias related to data classification. However, they often faced bias and lack of information and needed further clarifications from the authors of the included studies. The inter-rater agreement was not evaluated during the literature search, which also might impact negatively the quality of the results of the present Baysiean network meta-analysis.

## Conclusion

Within the considered physiotherapeutic and non-conventional approaches to manage nonspecific and/or mechanic cLBP, adapted physical exercise, physical agent modalities, and a multidisciplinary approach might represent the most effective strategy in reducing pain and disability.

### Supplementary Information

Supplementary Information.

## Data Availability

The datasets generated during and or analysed during the current study are available throughout the manuscript.

## References

[1] Balague F, Mannion AF, Pellise F, Cedraschi C (2012). Non-specific low back pain. Lancet.

[2] Hopayian K, Raslan E, Soliman S (2023). The association of modic changes and chronic low back pain: A systematic review. J. Orthop..

[3] Migliorini F, Maffulli N, Baroncini A, Eschweiler J, Tingart M, Quack V (2021). Opioids for chronic low back pain management: a Bayesian network meta-analysis. Expert Rev. Clin. Pharmacol..

[4] Hoy D, Brooks P, Blyth F, Buchbinder R (2010). The Epidemiology of low back pain. Best Pract. Res. Clin. Rheumatol..

[5] Hartvigsen J, Hancock MJ, Kongsted A, Louw Q, Ferreira ML, Genevay S, Hoy D, Karppinen J, Pransky G, Sieper J, Smeets RJ, Underwood M, Working Lancet Low Back Pain Series, G,  (2018). What low back pain is and why we need to pay attention. Lancet.

[6] Delpierre Y (2022). Fear-avoidance beliefs, anxiety and depression are associated with motor control and dynamics parameters in patients with chronic low back pain. J. Orthop..

[7] Migliorini F, Maffulli N, Eschweiler J, Betsch M, Catalano G, Driessen A, Tingart M, Baroncini A (2021). The pharmacological management of chronic lower back pain. Expert Opin. Pharmacother..

[8] Wang F, Sun R, Zhang SD, Wu XT (2023). Comparison of thoracolumbar versus non-thoracolumbar osteoporotic vertebral compression fractures in risk factors, vertebral compression degree and pre-hospital back pain. J. Orthop. Surg. Res..

[9] Pourahmadi M, Negahban H, Koes BW, Fernandez-de-Las-Penas C, Ebrahimi Takamjani I, Bahramian M (2023). The effect of dual-task conditions on postural control in adults with low back pain: a systematic review and meta-analysis. J. Orthop. Surg. Res..

[10] Lu W, Shen Z, Chen Y, Hu X, Ruan C, Ma W, Jiang W (2023). Risk factors analysis and risk prediction model construction of non-specific low back pain: an ambidirectional cohort study. J. Orthop. Surg. Res..

[11] Zhang SK, Gu ML, Zhang T, Xu H, Mao SJ, Zhou WS (2023). Effects of exercise therapy on disability, mobility, and quality of life in the elderly with chronic low back pain: a systematic review and meta-analysis of randomized controlled trials. J. Orthop. Surg. Res..

[12] Foster NE, Anema JR, Cherkin D, Chou R, Cohen SP, Gross DP, Ferreira PH, Fritz JM, Koes BW, Peul W, Turner JA, Maher CG, Working Lancet Low Back Pain Series, G,  (2018). Prevention and treatment of low back pain: evidence, challenges, and promising directions. Lancet.

[13] Qaseem, A., Wilt, T.J., McLean, R.M., Forciea, M.A., Clinical Guidelines Committee of the American College of P, Denberg, T.D., Barry, M.J., Boyd, C., Chow, R.D., Fitterman, N., Harris, R.P., Humphrey, L.L., Vijan, S. Noninvasive treatments for acute, subacute, and chronic low back pain: A clinical practice guideline from the american college of physicians. Ann. Intern. Med. 166 (7):514-530. 10.7326/M16-2367 (2017) 10.7326/M16-2367 28192789

[14] Baroncini A, Maffulli N, Eschweiler J, Knobe M, Tingart M, Migliorini F (2021). Management of facet joints osteoarthritis associated with chronic low back pain: A systematic review. Surgeon.

[15] Geneen LJ, Moore RA, Clarke C, Martin D, Colvin LA, Smith BH (2017). Physical activity and exercise for chronic pain in adults: an overview of Cochrane Reviews. Cochrane Database Syst. Rev..

[16] Riley DA, Van Dyke JM (2012). The effects of active and passive stretching on muscle length. Phys. Med. Rehabil. Clin. N. Am.

[17] Taylor NF, Dodd KJ, Shields N, Bruder A (2007). Therapeutic exercise in physiotherapy practice is beneficial: a summary of systematic reviews 2002–2005. Aust. J. Physiother..

[18] Czaplewski LG, Rimmer O, McHale D, Laslett M (2023). Modic changes as seen on MRI are associated with nonspecific chronic lower back pain and disability. J. Orthop. Surg. Res..

[19] Jia CQ, Wu YJ, Cao SQ, Hu FQ, Zheng ZR, Xu C, Zhang XS (2023). Mid-term low back pain improvement after total hip arthroplasty in 306 patients with developmental dysplasia of the hip. J. Orthop. Surg. Res..

[20] Liu K, Zhang Q, Chen L, Zhang H, Xu X, Yuan Z, Dong J (2023). Efficacy and safety of extracorporeal shockwave therapy in chronic low back pain: a systematic review and meta-analysis of 632 patients. J. Orthop. Surg. Res..

[21] Park HJ, Choi JY, Lee WM, Park SM (2023). Prevalence of chronic low back pain and its associated factors in the general population of South Korea: a cross-sectional study using the National Health and Nutrition Examination Surveys. J. Orthop. Surg. Res..

[22] Hernandez-Lucas P, Leiros-Rodriguez R, Lopez-Barreiro J, Garcia-Soidan JL (2022). Is the combination of exercise therapy and health education more effective than usual medical care in the prevention of non-specific back pain? A systematic review with meta-analysis. Ann. Med..

[23] Miranda L, Quaranta M, Oliva F, Maffulli N (2023). Stem cells and discogenic back pain. Br. Med. Bull..

[24] Migliorini F, Vaishya R, Pappalardo G, Schneider M, Bell A, Maffulli N (2023). Between guidelines and clinical trials: evidence-based advice on the pharmacological management of non-specific chronic low back pain. BMC Musculoskelet. Disord..

[25] Baroncini A, Maffulli N, Eschweiler J, Molsberger F, Klimuch A, Migliorini F (2022). Acupuncture in chronic aspecific low back pain: a Bayesian network meta-analysis. J. Orthop. Surg. Res..

[26] Oliveira CB, Maher CG, Pinto RZ, Traeger AC, Lin CC, Chenot JF, van Tulder M, Koes BW (2018). Clinical practice guidelines for the management of non-specific low back pain in primary care: an updated overview. Eur. Spine J..

[27] George SZ, Fritz JM, Silfies SP, Schneider MJ, Beneciuk JM, Lentz TA, Gilliam JR, Hendren S, Norman KS (2021). Interventions for the management of acute and chronic low back pain: Revision 2021. J. Orthop. Sports Phys. Ther..

[28] Fernandez-Rodriguez R, Alvarez-Bueno C, Cavero-Redondo I, Torres-Costoso A, Pozuelo-Carrascosa DP, Reina-Gutierrez S, Pascual-Morena C, Martinez-Vizcaino V (2022). Best exercise options for reducing pain and disability in adults with chronic low back pain: Pilates, strength, core-based, and mind-body. A network meta-analysis. J. Orthop. Sports Phys. Ther..

[29] Hayden JA, Ellis J, Ogilvie R, Malmivaara A, van Tulder MW (2021). Exercise therapy for chronic low back pain. Cochrane Database Syst. Rev..

[30] Grooten WJA, Bostrom C, Dedering A, Halvorsen M, Kuster RP, Nilsson-Wikmar L, Olsson CB, Rovner G, Tseli E, Rasmussen-Barr E (2022). Summarizing the effects of different exercise types in chronic low back pain—a systematic review of systematic reviews. BMC Musculoskelet. Disord..

[31] Tagliaferri SD, Mitchell UH, Saueressig T, Owen PJ, Miller CT, Belavy DL (2022). Classification approaches for treating low back pain have small effects that are not clinically meaningful: A systematic review with meta-analysis. J. Orthop. Sports Phys. Ther..

[32] Howick, J.C.I., Glasziou, P., Greenhalgh, T., Heneghan, C., Liberati, A., Moschetti, I., Phillips, B., Thornton, H., Goddard, O., Hodgkinson, M. The 2011 Oxford CEBM Levels of Evidence. Oxford Centre for Evidence-Based Medicine (2011). Available at http://www.cebmnet/indexaspx?o=5653

[33] Maher C, Underwood M, Buchbinder R (2017). Non-specific low back pain. Lancet.

[34] Borenstein D (2013). Mechanical low back pain–a rheumatologist's view. Nat. Rev. Rheumatol..

[35] Hutton B, Salanti G, Caldwell DM, Chaimani A, Schmid CH, Cameron C, Ioannidis JP, Straus S, Thorlund K, Jansen JP, Mulrow C, Catala-Lopez F, Gotzsche PC, Dickersin K, Boutron I, Altman DG, Moher D (2015). The PRISMA extension statement for reporting of systematic reviews incorporating network meta-analyses of health care interventions: checklist and explanations. Ann. Intern. Med..

[36] Abenhaim L, Rossignol M, Valat JP, Nordin M, Avouac B, Blotman F, Charlot J, Dreiser RL, Legrand E, Rozenberg S, Vautravers P (2000). The role of activity in the therapeutic management of back pain. Report of the International Paris Task Force on Back Pain. Spine (Phila Pa 1976).

[37] Hutzler Y, Sherrill C (2007). Defining adapted physical activity: international perspectives. Adapt. Phys. Activ. Q..

[38] Yurdakul OV, Beydogan E, Yilmaz Yalcinkaya E (2019). Effects of physical therapy agents on pain, disability, quality of life, and lumbar paravertebral muscle stiffness via elastography in patients with chronic low back pain. Turk. J. Phys. Med. Rehabil..

[39] O'Sullivan PB, Caneiro JP, O'Keeffe M, Smith A, Dankaerts W, Fersum K, O'Sullivan K (2018). Cognitive functional therapy: An integrated behavioral approach for the targeted management of disabling low back pain. Phys. Ther..

[40] Castro-Sanchez AM, Lara-Palomo IC, Mataran-Penarrocha GA, Fernandez-Sanchez M, Sanchez-Labraca N, Arroyo-Morales M (2012). Kinesio Taping reduces disability and pain slightly in chronic non-specific low back pain: a randomised trial. J. Physiother..

[41] Stevens ML, Lin CC, Maher CG (2016). The roland morris disability questionnaire. J. Physiother..

[42] Fairbank JC (2014). Oswestry disability index. J. Neurosurg. Spine.

[43] Hjermstad, M.J., Fayers, P.M., Haugen, D.F., Caraceni, A., Hanks, G.W., Loge, J.H., Fainsinger, R., Aass, N., Kaasa, S., European Palliative Care Research C Studies comparing Numerical Rating Scales, Verbal Rating Scales, and Visual Analogue Scales for assessment of pain intensity in adults: a systematic literature review. *J. Pain Symptom Manage.* 41(6):1073-1093 (2011). 10.1016/j.jpainsymman.2010.08.016 10.1016/j.jpainsymman.2010.08.016 21621130

[44] Cumpston, M., Li, T., Page, M.J., Chandler, J., Welch, V.A., Higgins, J.P., Thomas, J. Updated guidance for trusted systematic reviews: a new edition of the Cochrane Handbook for Systematic Reviews of Interventions. *Cochrane Database Syst. Rev.* 10, D000142 (2019). 10.1002/14651858.ED000142 10.1002/14651858.ED000142 PMC10284251 31643080

[45] Higgins, J.P.T.T.J., Chandler, J., Cumpston, M., Li, T., Page, M.J., Welch, V.A. Cochrane Handbook for Systematic Reviews of Interventions version 6.2. Cochrane 2021. Available from www.training.cochrane.org/handbook. Accessed on February 2022.

[46] Altman, D.G. London UCaHPSfMR, First Edition.

[47] Aasa B, Berglund L, Michaelson P, Aasa U (2015). Individualized low-load motor control exercises and education versus a high-load lifting exercise and education to improve activity, pain intensity, and physical performance in patients with low back pain: a randomized controlled trial. J. Ortho. Sports Phys. Therapy.

[48] Balthazard P, de Goumoens P, Rivier G, Demeulenaere P, Ballabeni P, Deriaz O (2012). Manual therapy followed by specific active exercises versus a placebo followed by specific active exercises on the improvement of functional disability in patients with chronic non specific low back pain: a randomized controlled trial. BMC Musculoskelet. Disord..

[49] Bhadauria, E.A., Gurudut, P. Comparative effectiveness of lumbar stabilization, dynamic strengthening, and Pilates on chronic low back pain: randomized clinical trial. *J. Exerc. Rehabil.* 13(4):477-485 (2017). 10.12965/jer.1734972.486 10.12965/jer.1734972.486 PMC5667628 29114516

[50] Cecchi F, Molino-Lova R, Chiti M, Pasquini G, Paperini A, Conti AA, Macchi C (2010). Spinal manipulation compared with back school and with individually delivered physiotherapy for the treatment of chronic low back pain: a randomized trial with one-year follow-up. Clin. Rehabil..

[51] Costa LOP, Maher CG, Latimer J, Hodges PW, Herbert RD, Refshauge KM, McAuley JH, Jennings MD (2009). Motor control exercise for chronic low back pain: A randomized placebo-controlled trial. Phys. Therapy.

[52] Vibe Fersum K, Smith A, Kvale A, Skouen JS, O'Sullivan P (2019). Cognitive functional therapy in patients with non-specific chronic low back pain-a randomized controlled trial 3-year follow-up. Eur. J. Pain.

[53] Garcia AN, Costa LdCM, da Silva TM, Gondo FLB, Cyrillo FN, Costa RA, Costa LOP (2013). Effectiveness of back school versus McKenzie exercises in patients with chronic nonspecific low back pain: a randomized controlled trial. Physical therapy.

[54] Goldby, L.J., Moore, A.P., Doust, J., Trew, M.E. A randomized controlled trial investigating the efficiency of musculoskeletal physiotherapy on chronic low back disorder. *Spine (Phila Pa 1976)* 31(10), 1083-1093 (2006). 10.1097/01.brs.0000216464.37504.64 10.1097/01.brs.0000216464.37504.64 16648741

[55] Halliday MH, Pappas E, Hancock MJ, Clare HA, Pinto RZ, Robertson G, Ferreira PH (2016). A randomized controlled trial comparing the mckenzie method to motor control exercises in people with chronic low back pain and a directional preference. J. Orthop. Sports Phys. Ther..

[56] Hohmann CD, Stange R, Steckhan N, Robens S, Ostermann T, Paetow A, Michalsen A (2018). The effectiveness of leech therapy in chronic low back pain. Dtsch. Arztebl. Int..

[57] Kaapa, E.H., Frantsi, K., Sarna, S., Malmivaara, A. Multidisciplinary group rehabilitation versus individual physiotherapy for chronic nonspecific low back pain: a randomized trial. *Spine (Phila Pa 1976)* 31(4), 371-376 (2006). 10.1097/01.brs.0000200104.90759.8c 10.1097/01.brs.0000200104.90759.8c 16481945

[58] Kobayashi D, Shimbo T, Hayashi H, Takahashi O (2019). Shiatsu for chronic lower back pain: randomized controlled study. Complement. Therap. Med..

[59] Lawand P, Lombardi Júnior I, Jones A, Sardim C, Ribeiro LH, Natour J (2015). Effect of a muscle stretching program using the global postural reeducation method for patients with chronic low back pain: A randomized controlled trial. Joint Bone Spine.

[60] Macedo LdB, Richards J, Borges DT, Melo SA, Brasileiro JS (2019). Kinesio Taping reduces pain and improves disability in low back pain patients: a randomised controlled trial. Physiotherapy.

[61] Majchrzycki M, Kocur P, Kotwicki T (2014). Deep tissue massage and nonsteroidal anti-inflammatory drugs for low back pain: a prospective randomized trial. ScientificWorldJournal.

[62] Murtezani A, Govori V, Meka VS, Ibraimi Z, Rrecaj S, Gashi S (2015). A comparison of mckenzie therapy with electrophysical agents for the treatment of work related low back pain: A randomized controlled trial. J. Back Musculoskelet. Rehabil..

[63] Sahin N, Karahan AY, Albayrak I (2018). Effectiveness of physical therapy and exercise on pain and functional status in patients with chronic low back pain: a randomized-controlled trial. Turk. J. Phys. Med. Rehabil..

[64] Saper RB, Lemaster C, Delitto A, Sherman KJ, Herman PM, Sadikova E, Stevans J, Keosaian JE, Cerrada CJ, Femia AL (2017). Yoga, physical therapy, or education for chronic low back pain: a randomized noninferiority trial. Annals Internal Med..

[65] Suh JH, Kim H, Jung GP, Ko JY, Ryu JS (2019). The effect of lumbar stabilization and walking exercises on chronic low back pain: A randomized controlled trial. Medicine (Baltimore).

[66] Takahashi N, Omata JI, Iwabuchi M, Fukuda H, Shirado O (2017). Therapeutic efficacy of nonsteroidal anti-inflammatory drug therapy versus exercise therapy in patients with chronic nonspecific low back pain: a prospective study. Fukushima J. Med. Sci..

[67] Uzunkulaoglu A, Gunes Aytekin M, Ay S, Ergin S (2018). The effectiveness of Kinesio taping on pain and clinical features in chronic non-specific low back pain: A randomized controlled clinical trial. Turk. J. Phys. Med. Rehabil..

[68] Yeung CK, Leung MC, Chow DH (2003). The use of electro-acupuncture in conjunction with exercise for the treatment of chronic low-back pain. J Altern Complement Med.

[69] Dufour, N., Thamsborg, G., Oefeldt, A., Lundsgaard, C., Stender, S. Treatment of chronic low back pain: a randomized, clinical trial comparing group-based multidisciplinary biopsychosocial rehabilitation and intensive individual therapist-assisted back muscle strengthening exercises. *Spine (Phila Pa 1976)* 35(5), 469-476 (2010). 10.1097/BRS.0b013e3181b8db2e 10.1097/BRS.0b013e3181b8db2e 20147878

[70] Helmhout P, Harts C, Staal J, Candel M, De Bie R (2004). Comparison of a high-intensity and a low-intensity lumbar extensor training program as minimal intervention treatment in low back pain: a randomized trial. Eur. Spine J..

[71] Jarzem PF, Harvey EJ, Arcaro N, Kaczorowski J (2005). Transcutaneous electrical nerve stimulation [TENS] for chronic low back pain. J. Musculoskelet. Pain.

[72] Meng K, Seekatz B, Roband H, Worringen U, Vogel H, Faller H (2011). Intermediate and long-term effects of a standardized back school for inpatient orthopedic rehabilitation on illness knowledge and self-management behaviors: a randomized controlled trial. Clin. J. Pain.

[73] Prommanon B, Puntumetakul R, Puengsuwan P, Chatchawan U, Kamolrat T, Rittitod T, Yamauchi J (2015). Effectiveness of a back care pillow as an adjuvant physical therapy for chronic non-specific low back pain treatment: a randomized controlled trial. J. Phys. Therapy Sci..

[74] Tavafian SS, Jamshidi AR, Mohammad K (2011). Treatment of chronic low back pain: a randomized clinical trial comparing multidisciplinary group-based rehabilitation program and oral drug treatment with oral drug treatment alone. Clin. J. Pain.

[75] Tavafian SS, Jamshidi AR, Mohammad K (2014). Treatment of low back pain: randomized clinical trial comparing a multidisciplinary group-based rehabilitation program with oral drug treatment up to 12 months. Int. J. Rheum. Dis..

[76] Alfuth M, Cornely D (2016). Chronic low back pain: Comparison of mobilization and core stability exercises. Der. Orthopäde.

[77] Grande-Alonso M, Suso-Martí L, Cuenca-Martínez F, Pardo-Montero J, Gil-Martínez A, La Touche R (2019). Physiotherapy based on a biobehavioral approach with or without orthopedic manual physical therapy in the treatment of nonspecific chronic low back pain: a randomized controlled trial. Pain Med..

[78] Ali MN, Sethi K, Noohu MM (2019). Comparison of two mobilization techniques in management of chronic non-specific low back pain. J. Bodywork Move. Therap..

[79] Ahmadi H, Adib H, Selk-Ghaffari M, Shafizad M, Moradi S, Madani Z, Partovi G, Mahmoodi A (2020). Comparison of the effects of the Feldenkrais method versus core stability exercise in the management of chronic low back pain: a randomised control trial. Clin. Rehabil..

[80] Almhdawi KA, Obeidat DS, Kanaan SF, Oteir AO, Mansour ZM, Alrabbaei H (2020). Efficacy of an innovative smartphone application for office workers with chronic non-specific low back pain: a pilot randomized controlled trial. Clin. Rehabil..

[81] Added MAN, Costa LOP, de Freitas DG, Fukuda TY, Monteiro RL, Salomão EC, de Medeiros FC, Costa LdCM (2016). Kinesio taping does not provide additional benefits in patients with chronic low back pain who receive exercise and manual therapy: a randomized controlled trial. J. Orthop. Sports Phys. Therapy.

[82] Arampatzis A, Schroll A, Catalá MM, Laube G, Schüler S, Dreinhofer K (2017). A random-perturbation therapy in chronic non-specific low-back pain patients: a randomised controlled trial. Eur. J. Appl. Physiol..

[83] Areeudomwong P, Wongrat W, Neammesri N, Thongsakul T (2017). A randomized controlled trial on the long-term effects of proprioceptive neuromuscular facilitation training, on pain-related outcomes and back muscle activity, in patients with chronic low back pain. Musculoskelet. Care.

[84] Bae C-R, Jin Y, Yoon B-C, Kim N-H, Park K-W, Lee S-H (2018). Effects of assisted sit-up exercise compared to core stabilization exercise on patients with non-specific low back pain: A randomized controlled trial. J. Back Musculoskelet. Rehabil..

[85] Bi X, Zhao J, Zhao L, Liu Z, Zhang J, Sun D, Song L, Xia Y (2013). Pelvic floor muscle exercise for chronic low back pain. J. Int. Med. Res..

[86] Bicalho E, Setti JA, Macagnan J, Cano JL, Manffra EF (2010). Immediate effects of a high-velocity spine manipulation in paraspinal muscles activity of nonspecific chronic low-back pain subjects. Man. Ther..

[87] Bronfort G, Maiers MJ, Evans RL, Schulz CA, Bracha Y, Svendsen KH, Grimm RH, Owens EF, Garvey TA, Transfeldt EE (2011). Supervised exercise, spinal manipulation, and home exercise for chronic low back pain: a randomized clinical trial. The Spine J..

[88] Cai, C., Yang, Y., Kong, P.W. Comparison of lower limb and back exercises for runners with chronic low back pain (2017). 10.1249/MSS.0000000000001396 28767525

[89] Azevedo DC, Ferreira PH, Santos HdO, Oliveira DR, de Souza JVL, Costa LOP (2018). Movement system impairment-based classification treatment versus general exercises for chronic low back pain: randomized controlled trial. Phys. Therapy.

[90] Castro-Sánchez AM, Lara-Palomo IC, Matarán-Peñarrocha GA, Fernández-De-Las-Peñas C, Saavedra-Hernández M, Cleland J, Aguilar-Ferrándiz ME (2016). Short-term effectiveness of spinal manipulative therapy versus functional technique in patients with chronic nonspecific low back pain: a pragmatic randomized controlled trial. The Spine J..

[91] Ryan CG, Gray HG, Newton M, Granat MH (2010). Pain biology education and exercise classes compared to pain biology education alone for individuals with chronic low back pain: a pilot randomised controlled trial. Man. Ther..

[92] Chhabra H, Sharma S, Verma S (2018). Smartphone app in self-management of chronic low back pain: a randomized controlled trial. Eur. Spine J..

[93] Cortell-Tormo JM, Sanchez PT, Chulvi-Medrano I, Tortosa-Martinez J, Manchado-Lopez C, Llana-Belloch S, Perez-Soriano P (2018). Effects of functional resistance training on fitness and quality of life in females with chronic nonspecific low-back pain. J. Back Musculoskelet. Rehabil..

[94] Cruz-Díaz D, Martínez-Amat A, Manuel J, Casuso RA, de Guevara NML, Hita-Contreras F (2015). Effects of a six-week Pilates intervention on balance and fear of falling in women aged over 65 with chronic low-back pain: A randomized controlled trial. Maturitas.

[95] Cruz-Díaz D, Bergamin M, Gobbo S, Martínez-Amat A, Hita-Contreras F (2017). Comparative effects of 12 weeks of equipment based and mat Pilates in patients with Chronic Low Back Pain on pain, function and transversus abdominis activation. A randomized controlled trial. Complement. Ther. Med..

[96] Cuesta-Vargas AI, Adams N (2011). A pragmatic community-based intervention of multimodal physiotherapy plus deep water running (DWR) for fibromyalgia syndrome: a pilot study. Clin. Rheumatol..

[97] Diab AAM, Moustafa IM (2013). The efficacy of lumbar extension traction for sagittal alignment in mechanical low back pain: a randomized trial. J. Back Musculoskelet. Rehabil..

[98] Koldaş Doğan Ş, Sonel Tur B, Kurtaiş Y, Atay MB (2008). Comparison of three different approaches in the treatment of chronic low back pain. Clin. Rheumatol..

[99] Eardley S, Brien S, Little P, Prescott P, Lewith G (2013). Professional kinesiology practice for chronic low back pain: single-blind, randomised controlled pilot study. Complement. Med. Res..

[100] Engbert, K., Weber, M. The effects of therapeutic climbing in patients with chronic low back pain: a randomized controlled study. *Spine (Phila Pa 1976)* 36(11), 842-849 (2011). 10.1097/BRS.0b013e3181e23cd1 10.1097/BRS.0b013e3181e23cd1 21192296

[101] de Oliveira RF, Liebano RE, Costa Lda C, Rissato LL, Costa LO (2013). Immediate effects of region-specific and non-region-specific spinal manipulative therapy in patients with chronic low back pain: a randomized controlled trial. Phys. Ther..

[102] Franca FR, Burke TN, Caffaro RR, Ramos LA, Marques AP (2012). Effects of muscular stretching and segmental stabilization on functional disability and pain in patients with chronic low back pain: a randomized, controlled trial. J Manip. Physiol. Ther..

[103] Friedrich M, Gittler G, Halberstadt Y, Cermak T, Heiller I (1998). Combined exercise and motivation program: Effect on the compliance and level of disability of patients with chronic low back pain: A randomized controlled trial. Arch. Phys. Med. Rehabil..

[104] Frost H, Klaber Moffett JA, Moser JS, Fairbank JC (1995). Randomised controlled trial for evaluation of fitness programme for patients with chronic low back pain. BMJ.

[105] Garcia AN, Costa LdCM, Hancock MJ, De Souza FS, de Oliveira Gomes GVF, De Almeida MO, Costa LOP (2018). McKenzie Method of Mechanical Diagnosis and Therapy was slightly more effective than placebo for pain, but not for disability, in patients with chronic non-specific low back pain: a randomised placebo controlled trial with short and longer term follow-up. Br. J. Sports Med..

[106] Gardner T, Refshauge K, McAuley J, Hübscher M, Goodall S, Smith L (2019). Combined education and patient-led goal setting intervention reduced chronic low back pain disability and intensity at 12 months: a randomised controlled trial. Br. J. Sports Med..

[107] Gavish L, Barzilay Y, Koren C, Stern A, Weinrauch L, Friedman D (2015). Novel continuous passive motion device for self-treatment of chronic lower back pain: a randomised controlled study. Physiotherapy.

[108] Geisser ME, Wiggert EA, Haig AJ, Colwell MO (2005). A randomized, controlled trial of manual therapy and specific adjuvant exercise for chronic low back pain. Clin. J. Pain.

[109] Gwon A-J, Kim S-Y, Oh D-W (2020). Effects of integrating Neurac vibration into a side-lying bridge exercise on a sling in patients with chronic low back pain: a randomized controlled study. Physiother. Theory Pr..

[110] Haas M, Vavrek D, Peterson D, Polissar N, Neradilek MB (2014). Dose-response and efficacy of spinal manipulation for care of chronic low back pain: a randomized controlled trial. The Spine J..

[111] Halliday MH, Pappas E, Hancock MJ, Clare HA, Pinto RZ, Robertson G, Ferreira PH (2019). A randomized clinical trial comparing the McKenzie method and motor control exercises in people with chronic low back pain and a directional preference: 1-year follow-up. Physiotherapy.

[112] Harts CC, Helmhout PH, de Bie RA, Staal JB (2008). A high-intensity lumbar extensor strengthening program is little better than a low-intensity program or a waiting list control group for chronic low back pain: a randomised clinical trial. Australian J. Physiother..

[113] Macedo LG, Maher CG, Hancock MJ, Kamper SJ, McAuley JH, Stanton TR, Stafford R, Hodges PW (2014). Predicting response to motor control exercises and graded activity for patients with low back pain: preplanned secondary analysis of a randomized controlled trial. Phys. Therapy.

[114] Javadian Y, Behtash H, Akbari M, Taghipour-Darzi M, Zekavat H (2012). The effects of stabilizing exercises on pain and disability of patients with lumbar segmental instability. J. Back Musculoskelet. Rehabil..

[115] Fagundes Loss J, de Souza da Silva, L., Ferreira Miranda, I., Groisman, S., Santiago Wagner Neto, E., Souza, C., Tarragô Candotti, C.  (2020). Immediate effects of a lumbar spine manipulation on pain sensitivity and postural control in individuals with nonspecific low back pain: a randomized controlled trial. Chiropr. Man. Ther..

[116] Kell RT, Risi AD, Barden JM (2011). The response of persons with chronic nonspecific low back pain to three different volumes of periodized musculoskeletal rehabilitation. J. Strength Cond. Res..

[117] Kim TH, Kim EH, Cho HY (2015). The effects of the CORE programme on pain at rest, movement-induced and secondary pain, active range of motion, and proprioception in female office workers with chronic low back pain: a randomized controlled trial. Clin. Rehabil..

[118] Kim YW, Kim NY, Chang WH, Lee SC (2018). Comparison of the therapeutic effects of a sling exercise and a traditional stabilizing exercise for clinical lumbar spinal instability. J. Sport Rehabil..

[119] Tekur P, Singphow C, Nagendra HR, Raghuram N (2008). Effect of short-term intensive yoga program on pain, functional disability and spinal flexibility in chronic low back pain: a randomized control study. The J. Altern. Complement. Med..

[120] Tekur P, Nagarathna R, Chametcha S, Hankey A, Nagendra H (2012). A comprehensive yoga programs improves pain, anxiety and depression in chronic low back pain patients more than exercise: an RCT. Complement. Ther. Med..

[121] de Oliveira RF, Costa LOP, Nascimento LP, Rissato LL (2020). Directed vertebral manipulation is not better than generic vertebral manipulation in patients with chronic low back pain: a randomised trial. J. Physiother..

[122] Zou L, Zhang Y, Liu Y, Tian X, Xiao T, Liu X, Yeung AS, Liu J, Wang X, Yang Q (2019). The Effects of Tai Chi Chuan versus core stability training on lower-limb neuromuscular function in aging individuals with non-specific chronic lower back pain. Medicina (Kaunas).

[123] Zhang Y, Wan L, Wang X (2014). The effect of health education in patients with chronic low back pain. J. Int. Med. Res..

[124] Zheng Z, Wang J, Gao Q, Hou J, Ma L, Jiang C, Chen G (2012). Therapeutic evaluation of lumbar tender point deep massage for chronic non-specific low back pain. J. Tradit. Chin. Med..

[125] Yang C-Y, Tsai Y-A, Wu P-K, Ho S-Y, Chou C-Y, Huang S-F (2021). Pilates-based core exercise improves health-related quality of life in people living with chronic low back pain: A pilot study. J. Bodywork Move. Ther..

[126] Waseem M, Karimi H, Gilani SA, Hassan D (2019). Treatment of disability associated with chronic non-specific low back pain using core stabilization exercises in Pakistani population. J. Back Musculoskelet. Rehabil..

[127] Williams KA, Petronis J, Smith D, Goodrich D, Wu J, Ravi N, Doyle EJ, Juckett RG, Kolar MM, Gross R (2005). Effect of Iyengar yoga therapy for chronic low back pain. Pain.

[128] Verbrugghe J, Hansen D, Demoulin C, Verbunt J, Roussel NA, Timmermans A (2021). High intensity training is an effective modality to improve long-term disability and exercise capacity in chronic nonspecific low back pain: a randomized controlled trial. Int. J. Environ. Res. Public Health.

[129] Sipaviciene S, Kliziene I (2020). Effect of different exercise programs on non-specific chronic low back pain and disability in people who perform sedentary work. Clin. Biomech..

[130] Phattharasupharerk S, Purepong N, Eksakulkla S, Siriphorn A (2019). Effects of Qigong practice in office workers with chronic non-specific low back pain: a randomized control trial. J. Bodywork Move. Ther..

[131] Magalhães MO, Comachio J, Ferreira PH, Pappas E, Marques AP (2018). Effectiveness of graded activity versus physiotherapy in patients with chronic nonspecific low back pain: midterm follow up results of a randomized controlled trial. Br. J. Phys. Ther..

[132] Monticone M, Ferrante S, Rocca B, Baiardi P, Dal Farra F, Foti C (2013). Effect of a long-lasting multidisciplinary program on disability and fear-avoidance behaviors in patients with chronic low back pain: results of a randomized controlled trial. Clin. J. Pain.

[133] Monticone M, Ambrosini E, Rocca B, Magni S, Brivio F, Ferrante S (2014). A multidisciplinary rehabilitation programme improves disability, kinesiophobia and walking ability in subjects with chronic low back pain: results of a randomised controlled pilot study. Eur. Spine J..

[134] Morone G, Paolucci T, Alcuri MR, Vulpiani MC, Matano A, Bureca I, Paolucci S, Saraceni VM (2011). Quality of life improved by multidisciplinary back school program in patients with chronic non-specific low back pain: a single blind randomized controlled trial. Eur. J. Phys. Rehabil. Med..

[135] Matarán-Peñarrocha GA, Lara Palomo IC, Antequera Soler E, Gil-Martínez E, Fernández-Sánchez M, Aguilar-Ferrándiz ME, Castro-Sánchez AM (2020). Comparison of efficacy of a supervised versus non-supervised physical therapy exercise program on the pain, functionality and quality of life of patients with non-specific chronic low-back pain: a randomized controlled trial. Clin. Rehabil..

[136] Laosee O, Sritoomma N, Wamontree P, Rattanapan C, Sitthi-Amorn C (2020). The effectiveness of traditional Thai massage versus massage with herbal compress among elderly patients with low back pain: A randomised controlled trial. Complement. Ther. Med..

[137] Rittweger, J., Just, K., Kautzsch, K., Reeg, P., Felsenberg, D. Treatment of chronic lower back pain with lumbar extension and whole-body vibration exercise: a randomized controlled trial. LWW (2002) 10.1097/00007632-200209010-00003 12221343

[138] Prado, É.R.A., Meireles, S.M., Carvalho, A.C.A., Mazoca, M.F., Motta Neto, A.D.M., Barboza Da Silva, R., Trindade Filho, E.M., Lombardi Junior, I., Natour, J. Influence of isostretching on patients with chronic low back pain. A randomized controlled trial. *Physiother. Theory Pr.* 37(2):287-294 (2021) 10.1080/09593985.2019.1625091 31161855

[139] Vollenbroek-Hutten MM, Hermens HJ, Wever D, Gorter M, Rinket J, IJzerman MJ,  (2004). Differences in outcome of a multidisciplinary treatment between subgroups of chronic low back pain patients defined using two multiaxial assessment instruments: the multidimensional pain inventory and lumbar dynamometry. Clin. Rehabil..

[140] del Pozo-Cruz B, Hernandez Mocholi MA, Adsuar JC, Parraca JA, Muro I, Gusi N (2011). Effects of whole body vibration therapy on main outcome measures for chronic non-specific low back pain: a single-blind randomized controlled trial. J. Rehabil. Med..

[141] Kostadinović S, Milovanović N, Jovanović J, Tomašević-Todorović S (2020). Efficacy of the lumbar stabilization and thoracic mobilization exercise program on pain intensity and functional disability reduction in chronic low back pain patients with lumbar radiculopathy: A randomized controlled trial. J. Back Musculoskelet. Rehabil..

[142] Monticone M, Ambrosini E, Rocca B, Cazzaniga D, Liquori V, Foti C (2016). Group-based task-oriented exercises aimed at managing kinesiophobia improved disability in chronic low back pain. Eur. J. Pain.

[143] Járomi M, Kukla A, Szilágyi B, Simon-Ugron Á, Bobály VK, Makai A, Linek P, Ács P, Leidecker E (2018). Back School programme for nurses has reduced low back pain levels: A randomised controlled trial. J. Clin. Nurs..

[144] Liu J, Yeung A, Xiao T, Tian X, Kong Z, Zou L, Wang X (2019). Chen-style tai chi for individuals (aged 50 years old or above) with chronic non-specific low back pain: a randomized controlled trial. Int. J. Environ. Res. Public Health.

[145] Lara-Palomo IC, Aguilar-Ferrándiz ME, Matarán-Peñarrocha GA, Saavedra-Hernández M, Granero-Molina J, Fernández-Sola C, Castro-Sánchez AM (2013). Short-term effects of interferential current electro-massage in adults with chronic non-specific low back pain: a randomized controlled trial. Clin. Rehabil..

[146] Saha FJ, Brummer G, Lauche R, Ostermann T, Choi K-E, Rampp T, Dobos G, Cramer H (2019). Gua Sha therapy for chronic low back pain: a randomized controlled trial. Complement. Therap. Clin. Pr..

[147] Segal-Snir Y, Lubetzky VA, Masharawi Y (2016). Rotation exercise classes did not improve function in women with non-specific chronic low back pain: A randomized single blind controlled study. J. Back Musculoskelet. Rehabil..

[148] Nambi GS, Inbasekaran D, Khuman R, Devi S, Jagannathan K (2014). Changes in pain intensity and health related quality of life with Iyengar yoga in nonspecific chronic low back pain: a randomized controlled study. Int. J. Yoga.

[149] Salamat S, Talebian S, Bagheri H, Maroufi N, Shaterzadeh MJ, Kalbasi G, O'Sullivan K (2017). Effect of movement control and stabilization exercises in people with extension related non-specific low back pain-a pilot study. J. Bodywork Move. Therap..

[150] Salavati M, Akhbari B, Takamjani IE, Bagheri H, Ezzati K, Kahlaee AH (2016). Effect of spinal stabilization exercise on dynamic postural control and visual dependency in subjects with chronic non-specific low back pain. J. Bodywork Move. Therap..

[151] Masharawi Y, Nadaf N (2013). The effect of non-weight bearing group-exercising on females with non-specific chronic low back pain: a randomized single blind controlled pilot study. J. Back Musculoskelet. Rehabil..

[152] Natour J, Cazotti LdA, Ribeiro LH, Baptista AS, Jones A (2015). Pilates improves pain, function and quality of life in patients with chronic low back pain: a randomized controlled trial. Clin. Rehabil..

[153] Murtezani A, Hundozi H, Orovcanec N, Sllamniku S, Osmani T (2011). A comparison of high intensity aerobic exercise and passive modalities for the treatment of workers with chronic low back pain: a randomized, controlled trial. Eur. J. Phys. Rehabil. Med..

[154] Kogure A, Kotani K, Katada S, Takagi H, Kamikozuru M, Isaji T, Hakata S (2015). A randomized, single-blind, placebo-controlled study on the efficacy of the arthrokinematic approach-hakata method in patients with chronic nonspecific low back pain. PLoS One.

[155] Ozsoy G, Ilcin N, Ozsoy I, Gurpinar B, Buyukturan O, Buyukturan B, Kararti C, Sas S (2019). The effects of myofascial release technique combined with core stabilization exercise in elderly with non-specific low back pain: a randomized controlled, single-blind study. Clin. Interv. Aging.

[156] Jousset N, Fanello S, Bontoux L, Dubus V, Billabert C, Vielle B, Roquelaure Y, Penneau-Fontbonne D, Richard I (2004). Effects of functional restoration versus 3 hours per week physical therapy: a randomized controlled study. Spine.

[157] Roche-Leboucher G, Petit-Lemanac'h A, Bontoux L, Dubus-Bausière V, Parot-Shinkel E, Fanello S, Penneau-Fontbonne D, Fouquet N, Legrand E, Roquelaure Y (2011). Multidisciplinary intensive functional restoration versus outpatient active physiotherapy in chronic low back pain: a randomized controlled trial. Spine.

[158] Khalil TM, Asfour SS, Martinez LM, Waly SM, Rosomoff RS, Rosomoff HL (1992). Stretching in the rehabilitation of low-back pain patients. Spine.

[159] Mannion AF, Muntener M, Taimela S, Dvorak J (1999). A randomized clinical trial of three active therapies for chronic low back pain. Spine.

[160] Mannion A, Müntener M, Taimela S, Dvorak J (2001). Comparison of three active therapies for chronic low back pain: results of a randomized clinical trial with one-year follow-up. Rheumatology.

[161] Yoon Y-S, Yu K-P, Lee KJ, Kwak S-H, Kim JY (2012). Development and application of a newly designed massage instrument for deep cross-friction massage in chronic non-specific low back pain. Annals Rehabil. Med..

[162] Yang J, Wei Q, Ge Y, Meng L, Zhao M (2019). Smartphone-based remote self-management of chronic low back pain: A preliminary study. J. Healthc. Eng..

[163] Hicks GE, Sions JM, Velasco TO, Manal TJ (2016). Trunk muscle training augmented with neuromuscular electrical stimulation appears to improve function in older adults with chronic low back pain: A randomized preliminary trial. Clin. J. Pain.

[164] Yalfani A, Raeisi Z, Koumasian Z (2020). Effects of eight-week water versus mat pilates on female patients with chronic nonspecific low back pain: Double-blind randomized clinical trial. J. Bodywork Move. Ther..

[165] Trapp W, Weinberger M, Erk S, Fuchs B, Mueller M, Gallhofer B, Hajak G, Kübler A, Lautenbacher S (2015). A brief intervention utilising visual feedback reduces pain and enhances tactile acuity in CLBP patients. J. Back Musculoskelet. Rehabil..

[166] Kofotolis N, Kellis E, Vlachopoulos SP, Gouitas I, Theodorakis Y (2016). Effects of Pilates and trunk strengthening exercises on health-related quality of life in women with chronic low back pain. J. Back Musculoskelet. Rehabil..

[167] Kuvacic G, Fratini P, Padulo J, Antonio DI, De Giorgio A (2018). Effectiveness of yoga and educational intervention on disability, anxiety, depression, and pain in people with CLBP: A randomized controlled trial. Complement. Ther. Clin. Pract..

[168] Hernandez-Reif M, Field T, Krasnegor J, Theakston H (2001). Lower back pain is reduced and range of motion increased after massage therapy. Int. J. Neurosci..

[169] Lewis, J. S., Hewitt, J. S., Billington, L., Cole, S., Byng, J., & Karayiannis, S. A randomized clinical trial comparing two physiotherapy interventions for chronic low back pain (2005). 10.1097/01.brs.0000157469.27779.de 15803071

[170] O'Keeffe M, O'Sullivan P, Purtill H, Bargary N, O'Sullivan K (2020). Cognitive functional therapy compared with a group-based exercise and education intervention for chronic low back pain: a multicentre randomised controlled trial (RCT). Br. J. Sports Med..

[171] Kaeding TS, Karch A, Schwarz R, Flor T, Wittke TC, Kück M, Böselt G, Tegtbur U, Stein L (2017). Whole-body vibration training as a workplace-based sports activity for employees with chronic low-back pain. Scand. J. Med. Sci. Sports.

[172] Petrozzi MJ, Leaver A, Ferreira PH, Rubinstein SM, Jones MK, Mackey MG (2019). Addition of MoodGYM to physical treatments for chronic low back pain: a randomized controlled trial. Chiropr. Man. Therap..

[173] Winter S (2015). Effectiveness of targeted home-based hip exercises in individuals with non-specific chronic or recurrent low back pain with reduced hip mobility: A randomised trial. J. Back Musculoskelet. Rehabil..

[174] Masse-Alarie H, Beaulieu LD, Preuss R, Schneider C (2017). Repetitive peripheral magnetic neurostimulation of multifidus muscles combined with motor training influences spine motor control and chronic low back pain. Clin. Neurophysiol..

[175] Martí-Salvador M, Hidalgo-Moreno L, Doménech-Fernández J, Lisón JF, Arguisuelas MD (2018). Osteopathic manipulative treatment including specific diaphragm techniques improves pain and disability in chronic nonspecific low back pain: a randomized trial. Arch. Phys. Med. Rehabil..

[176] Aguilar-Ferrándiz ME, Matarán-Peñarrocha GA, Tapia-Haro RM, Castellote-Caballero Y, Martí-García C, Castro-Sánchez AM (2022). Effects of a supervised exercise program in addition to electrical stimulation or kinesio taping in low back pain: a randomized controlled trial. Sci. Rep..

[177] Elgendy MH, Mohamed M, Hussein HM (2022). A single-blind randomized controlled trial investigating changes in electrical muscle activity, pain, and function after shockwave therapy in chronic non-specific low back pain: pilot study. Ortop. Traumatol. Rehabil..

[178] Fukuda TY, Aquino LM, Pereira P, Ayres I, Feio AF, de Jesus FLA, Neto MG (2021). Does adding hip strengthening exercises to manual therapy and segmental stabilization improve outcomes in patients with nonspecific low back pain? A randomized controlled trial. Braz. J. Phys. Ther..

[179] Ma KL, Zhao P, Cao CF, Luan FJ, Liao J, Wang QB, Fu ZH, Varrassi G, Wang HQ, Huang W (2021). Fus subcutaneous needling versus massage for chronic non-specific low-back pain: a randomized controlled clinical trial. Ann. Palliat. Med..

[180] Maggi, L., Celletti, C., Mazzarini, M., Blow, D., Camerota, F. Neuromuscular taping for chronic non-specific low back pain: a randomized single-blind controlled trial. *Aging Clin. Exp. Res.* 1-7 (2022). 10.1007/s40520-021-02029-0 34988932

[181] Jalalvandi F, Ghasemi R, Mirzaei M, Shamsi M (2022). Effects of back exercises versus transcutaneous electric nerve stimulation on relief of pain and disability in operating room nurses with chronic non-specific LBP: a randomized clinical trial. BMC Musculoskelet. Dis..

[182] Atilgan ED, Tuncer A (2021). The effects of breathing exercises in mothers of children with special health care needs: A randomized controlled trial. J. Back Musculoskelet. Rehabil..

[183] Pivovarsky MLF, Gaideski F, Macedo RM, Korelo RIG, Guarita-Souza LC, Liebano RE, Macedo ACB (2021). Immediate analgesic effect of two modes of transcutaneous electrical nerve stimulation on patients with chronic low back pain: a randomized controlled trial. Einstein (Sao Paulo).

[184] Van Dillen LR, Lanier VM, Steger-May K, Wallendorf M, Norton BJ, Civello JM, Czuppon SL, Francois SJ, Roles K, Lang CE (2021). Effect of motor skill training in functional activities vs strength and flexibility exercise on function in people with chronic low back pain: a randomized clinical trial. JAMA Neurol..

[185] Vibe Fersum K, O'Sullivan P, Skouen J, Smith A, Kvåle A (2013). Efficacy of classification-based cognitive functional therapy in patients with non-specific chronic low back pain: A randomized controlled trial. Eur. J. Pain.

[186] Ghroubi, S., Elleuch, H., Baklouti, S., Elleuch, M.-H. Les lombalgiques chroniques et manipulations vertébrales. Étude prospective à propos de 64 cas. In: Annales de réadaptation et de médecine physique, vol 7. Elsevier, pp 570-576 (2007). 10.1016/j.annrmp.2007.02.012 17382426

[187] Huber D, Grafetstätter C, Proßegger J, Pichler C, Wöll E, Fischer M, Dürl M, Geiersperger K, Höcketstaller M, Frischhut S (2019). Green exercise and mg-ca-SO4 thermal balneotherapy for the treatment of non-specific chronic low back pain: a randomized controlled clinical trial. BMC Musculoskelet. Dis..

[188] Werners R, Pynsent PB, Bulstrode CJ (1999). Randomized trial comparing interferential therapy with motorized lumbar traction and massage in the management of low back pain in a primary care setting. Spine (Phila Pa 1976).

[189] Kankaanpää M, Taimela S, Airaksinen O, Hänninen O (1999). The efficacy of active rehabilitation in chronic low back pain: effect on pain intensity, self-experienced disability, and lumbar fatigability. Spine.

[190] Marshall PW, Murphy BA (2008). Muscle activation changes after exercise rehabilitation for chronic low back pain. Arch. Phys. Med. Rehabil..

[191] Branchini M, Lopopolo F, Andreoli E, Loreti I, Marchand AM, Stecco A (2015). Fascial Manipulation® for chronic aspecific low back pain: a single blinded randomized controlled trial. F1000Research.

[192] Batıbay S, Külcü DG, Kaleoğlu Ö, Mesci N (2021). Effect of Pilates mat exercise and home exercise programs on pain, functional level, and core muscle thickness in women with chronic low back pain. J. Orthop. Sci..

[193] Elabd AM, Rasslan S-EB, Elhafez HM, Elabd OM, Behiry MA, Elerian AI (2020). Efficacy of integrating cervical posture correction with lumbar stabilization exercises for mechanical low back pain: a randomized blinded clinical trial. J. Appl. Biomech..

[194] Dadarkhah A, Rezaimoghadam F, Najafi S, Mohebi B, Azarakhsh A, Rezasoltani Z (2021). Remote versus in-person exercise instruction for chronic nonspecific low back pain lasting 12 weeks or longer: a randomized clinical trial. J. Natl. Med. Assoc..

[195] Nardin DMK, Stocco MR, Aguiar AF, Machado FA, de Oliveira RG, Andraus RAC (2022). Effects of photobiomodulation and deep water running in patients with chronic non-specific low back pain: a randomized controlled trial. Lasers Med. Sci..

[196] Owen PJ, Miller CT, Mundell NL, Verswijveren S, Tagliaferri SD, Brisby H, Bowe SJ, Belavy DL (2020). Which specific modes of exercise training are most effective for treating low back pain? Network meta-analysis. Br. J. Sports Med..

[197] Hayden JA, Ellis J, Ogilvie R, Stewart SA, Bagg MK, Stanojevic S, Yamato TP, Saragiotto BT (2021). Some types of exercise are more effective than others in people with chronic low back pain: a network meta-analysis. J. Physiother..

[198] Alhakami AM, Davis S, Qasheesh M, Shaphe A, Chahal A (2019). Effects of McKenzie and stabilization exercises in reducing pain intensity and functional disability in individuals with nonspecific chronic low back pain: a systematic review. J. Phys. Ther. Sci..

[199] de Campos TF, Maher CG, Clare HA, da Silva TM, Hancock MJ (2017). Effectiveness of McKenzie method-based self-management approach for the secondary prevention of a recurrence of low back pain (SAFE Trial): Protocol for a pragmatic randomized controlled trial. Phys. Ther..

[200] Lam OT, Strenger DM, Chan-Fee M, Pham PT, Preuss RA, Robbins SM (2018). Effectiveness of the McKenzie method of mechanical diagnosis and therapy for treating low back pain: Literature review with meta-analysis. J. Orthop. Sports Phys. Ther..

[201] Marshall A, Joyce CT, Tseng B, Gerlovin H, Yeh GY, Sherman KJ, Saper RB, Roseen EJ (2022). Changes in pain self-efficacy, coping skills, and fear-avoidance beliefs in a randomized controlled trial of yoga, physical therapy, and education for chronic low back pain. Pain. Med..

[202] Namnaqani FI, Mashabi AS, Yaseen KM, Alshehri MA (2019). The effectiveness of McKenzie method compared to manual therapy for treating chronic low back pain: a systematic review. J. Musculoskelet. Neuronal Interact..

[203] Patti A, Bianco A, Paoli A, Messina G, Montalto MA, Bellafiore M, Battaglia G, Iovane A, Palma A (2015). Effects of Pilates exercise programs in people with chronic low back pain: a systematic review. Medicine (Baltimore).

[204] Cimarras-Otal C, Marcen-Cinca N, Rabal-Pelay J, Lacrcel-Tejero B, Alczar-Crevilln A, Villalba-Ruete J, Bataller-Cervero AV (2020). Adapted exercises versus general exercise recommendations on chronic low back pain in industrial workers: A randomized control pilot study. Work.

[205] Marini S, Leoni E, Raggi A, Sanna T, Malavolta N, Angela B, Maietta Latessa P, Dallolio L (2019). Proposal of an adapted physical activity exercise protocol for women with osteoporosis-related vertebral fractures: A pilot study to evaluate feasibility, safety, and effectiveness. Int. J. Environ. Res. Public Health.

[206] Kuhnow A, Kuhnow J, Ham D, Rosedale R (2021). The McKenzie Method and its association with psychosocial outcomes in low back pain: a systematic review. Physiother. Theory Pract..

[207] Domingues de Freitas C, Costa DA, Junior NC, Civile VT (2020). Effects of the pilates method on kinesiophobia associated with chronic non-specific low back pain: Systematic review and meta-analysis. J. Bodyw. Mov. Ther..

[208] Jadhakhan F, Sobeih R, Falla D (2023). Effects of exercise/physical activity on fear of movement in people with spine-related pain: a systematic review. Front. Psychol..

[209] Snelgrove S, Liossi C (2013). Living with chronic low back pain: a metasynthesis of qualitative research. Chronic. Illn..

[210] Norlund A, Ropponen A, Alexanderson K (2009). Multidisciplinary interventions: review of studies of return to work after rehabilitation for low back pain. J. Rehabil. Med..

[211] Kamper SJ, Apeldoorn AT, Chiarotto A, Smeets RJ, Ostelo RW, Guzman J, van Tulder MW (2015). Multidisciplinary biopsychosocial rehabilitation for chronic low back pain: Cochrane systematic review and meta-analysis. BMJ.

[212] Heitz CA, Hilfiker R, Bachmann LM, Joronen H, Lorenz T, Uebelhart D, Klipstein A, Brunner F (2009). Comparison of risk factors predicting return to work between patients with subacute and chronic non-specific low back pain: systematic review. Eur. Spine J..

[213] Depreitere B, Jonckheer P, Coeckelberghs E, Desomer A, van Wambeke P (2020). The pivotal role for the multidisciplinary approach at all phases and at all levels in the national pathway for the management of low back pain and radicular pain in Belgium. Eur. J. Phys. Rehabil. Med..

[214] Peterson K, Anderson J, Bourne D, Mackey K, Helfand M (2018). Effectiveness of models used to deliver multimodal care for chronic musculoskeletal pain: A rapid evidence review. J. Gen. Intern. Med..

[215] Reese C, Mittag O (2013). Psychological interventions in the rehabilitation of patients with chronic low back pain: evidence and recommendations from systematic reviews and guidelines. Int. J. Rehabil. Res..

[216] van Erp RMA, Huijnen IPJ, Jakobs MLG, Kleijnen J, Smeets R (2019). Effectiveness of primary care interventions using a biopsychosocial approach in chronic low back pain: A systematic review. Pain Pract..

[217] van Middelkoop M, Rubinstein SM, Kuijpers T, Verhagen AP, Ostelo R, Koes BW, van Tulder MW (2011). A systematic review on the effectiveness of physical and rehabilitation interventions for chronic non-specific low back pain. Eur. Spine J..

