# Supplementary material for: Physiotherapeutic and non-conventional approaches in patients with chronic low-back pain: a level I Bayesian network meta-analysis
Source: Sci Rep. 2024 May 21;14:11546. doi: 10.1038/s41598-024-62276-9 (PMC11109223; doi:10.1038/s41598-024-62276-9)
Supplement: Supplementary file 1 — Supplementary Information. [file 41598_2024_62276_MOESM1_ESM.docx]

**Research Question:**

**Physiotherapeutic approaches in patients with chronic low-back pain: a level I systematic review**

**Concept 1: Disease**

**Keywords:**

Chronic low back pain

cLBP

non-specific low back pain

mechanical low back pain

chronic low back pain

**Mesh:**

"Low Back Pain"[Mesh]

"Low Back Pain/therapy"[Mesh]

"Low Back Pain/rehabilitation"[Mesh]

**Concept 2: Therapy**

**Keywords:**

Physiotherapy

Physical therapy

Manual therapy

Therapeutic Exercise (TE)

Adapted Physical Exercise (APE) Adaptive Training Exercise/Complementary Medicine (CM),

Manual Therapy (MT)

Physical Therapy (PT)

Education, Cognitive Re-education (CR)

Multidisciplinarity, Kinesiotaping (KT)

Sham Therapy (ST)

No Intervention

Massage

Mobilization

Spinal manipulation

Lumbar stabilization

Active and passive stretching

Exercises

Muscle exercises

Motor control exercises

Strengthening exercises

Stabilizing exercises

Functional resistance training

Muscle strength training

Back school

McKenzie

Acupuncture

Yoga

**Mesh:**

"Physical Therapy Modalities"[Mesh]

"Transcutaneous Electric Nerve Stimulation"[Mesh]

"Musculoskeletal Manipulations"[Mesh]

"Massage"[Mesh]

"Manipulation, Spinal"[Mesh]

"Muscle Stretching Exercises"[Mesh]

"Exercise"[Mesh]

"Exercise Therapy"[Mesh]

"Acupuncture"[Mesh]

"Acupuncture Therapy"[Mesh]

"Yoga"[Mesh]

**Searching Strategy**

"Low Back Pain"[Mesh] OR "Low Back Pain/therapy"[Mesh] OR "Low Back Pain/rehabilitation"[Mesh] OR "Spine"[Mesh] OR Chronic low back pain OR cLBP OR non-specific Low back pain OR mechanical Low back pain OR chronic Low back pain

**AND**

"Physical Therapy Modalities"[Mesh] OR physiotherapy

**AND**

"Musculoskeletal Manipulations"[Mesh] OR "Massage"[Mesh] OR "Manipulation, Spinal"[Mesh] OR "Muscle Stretching Exercises"[Mesh] OR "Exercise"[Mesh] OR "Exercise Therapy"[Mesh] OR "Acupuncture"[Mesh] OR "Acupuncture Therapy"[Mesh] OR "Yoga"[Mesh] OR physical therapy OR manual therapy OR Therapeutic Exercise OR TE OR Adapted Physical Exercise OR APE OR Adaptive Training Exercise/Complementary OR Medicine OR CM OR Manual Therapy OR MT OR Physical Therapy OR PT OR Education OR Cognitive Re-education OR CR OR Multidisciplinarity, Kinesiotaping OR KT OR Sham Therapy OR ST OR No Intervention OR massage OR mobilization OR spinal manipulation OR lumbar stabilization OR active and passive stretching OR exercises OR muscle exercises OR motor control exercises OR strengthening exercises OR stabilizing exercises OR functional resistance training OR muscle strength training OR Back school OR McKenzie OR Acupuncture OR Pilates OR Yoga

**SUMMARY**

(("Low Back Pain"[Mesh] OR "Low Back Pain/therapy"[Mesh] OR "Low Back Pain/rehabilitation"[Mesh] OR "Spine"[Mesh] OR Chronic low back pain OR cLBP OR non-specific Low back pain OR mechanical Low back pain OR chronic Low back pain) AND ("Physical Therapy Modalities"[Mesh] OR physiotherapy)) AND ("Musculoskeletal Manipulations"[Mesh] OR "Massage"[Mesh] OR "Manipulation, Spinal"[Mesh] OR "Muscle Stretching Exercises"[Mesh] OR "Exercise"[Mesh] OR "Exercise Therapy"[Mesh] OR "Acupuncture"[Mesh] OR "Acupuncture Therapy"[Mesh] OR "Yoga"[Mesh] OR physical therapy OR manual therapy OR Therapeutic Exercise OR TE OR Adapted Physical Exercise OR APE OR Adaptive Training Exercise/Complementary OR Medicine OR CM OR Manual Therapy OR MT OR Physical Therapy OR PT OR Education OR Cognitive Re-education OR CR OR Multidisciplinarity, Kinesiotaping OR KT OR Sham Therapy OR ST OR No Intervention OR massage OR mobilization OR spinal manipulation OR lumbar stabilization OR active and passive stretching OR exercises OR muscle exercises OR motor control exercises OR strengthening exercises OR stabilizing exercises OR functional resistance training OR muscle strength training OR Back school OR McKenzie OR Acupuncture OR Pilates OR Yoga)
